# Human Nervous System‐Based Biohybrid Robot‐On‐A‐Chip with Sensing Function for Toxicity Screening

**DOI:** 10.1002/advs.202501452

**Published:** 2025-07-02

**Authors:** Minkyu Shin, Joungpyo Lim, Seewoo Kim, Sangeun Lee, Wei Wen Su, Jinho Yoon, Jeong‐Woo Choi

**Affiliations:** ^1^ Department of Chemical & Biomolecular Engineering Sogang University 35 Baekbeom‐ro, Mapo‐gu Seoul 04107 Republic of Korea; ^2^ Department of Molecular Biosciences and Bioengineering University of Hawaii at Manoa Honolulu HI 96822 USA; ^3^ Department of Biomedical‐Chemical Engineering The Catholic University of Korea 43 Jibong‐ro, Wonmi‐gu Bucheon‐si Gyeonggi‐do 14662 Republic of Korea

**Keywords:** Biohybrid robot‐on‐a‐chip, Brain organoid, Eye assembloid, Human nervous system, Motor neuron spheroids, Muscle bundle, Toxicity screening

## Abstract

Biohybrid robots and biohybrid robot‐on‐a‐chip have been developed for drug screening and toxicity screening the complementing animal experiments. A sensing system is needed to evaluate the response to external stimulation, but until now, only the human motor system‐based biohybrid robot‐on‐a‐chip with neuromuscular system has been developed without a sensing system. A human nervous system‐based biohybrid robot‐on‐a‐chip with eye function as a sensing system in addition to brain/motor neuron/muscle functions is proposed for the first time. Eye assembloid is fabricated by a combination of thalamic organoid covered with Au nanomesh and four retinal organoids. By a combination of eye assembloid, cerebral organoid, motor neuron spheroid, and muscle bundle on polymer substrate, a human nervous system‐based biohybrid robot‐on‐a‐chip is made. When blue light is used to cause retinal damage or a hydroxychloroquine (HCQ), a retinal toxic chemical, is applied to the eye assembloid, they caused a decrease in muscle bundle contraction. These results indicated that electrophysiological signals generated by the eye assembloid are transmitted to the muscle bundle through cerebral organoid and motor neuron spheroids, and thus the proposed system can perform the toxicity screening. Human nervous system‐based biohybrid robot‐on‐a‐chip can be applied to drug screening of neurodegenerative diseases and toxicity screening for the complement of animal experiments in the future.

## Graphical abstract

Human nervous system‐based biohybrid robot‐on‐a chip with eye function as sensing system in addition to brain/motor neuron/muscle functions is proposed. Upon light stimulation, the eye assembloid generates electrophysiological signals, which are transmitted through the cerebral organoid and motor neuron spheroid, inducing muscle movement. This system can be applied to evaluate drugs targeting eye diseases, neurodegenerative diseases, and muscle disorders.

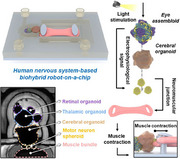

## 1 Introduction

Biohybrid robots, composed of tissue‐engineered biological materials, such as organoids, neural spheroids, and muscle bundles, have garnered significant attention due to their wide range of potential applications in the biomedical field.^[^ ^1^, ^2^, ^3^ ^]^ These systems have been applied in drug screening, disease modeling, and as ethical alternatives to traditional animal testing. More recently, with the growing emphasis on regenerative medicine, biohybrid robots have also been explored for neural and tissue regeneration by integrating soft materials, living cells, and external actuation mechanisms.^[^ ^4^, ^5^, ^6^ ^]^ These approaches not only promote tissue repair but also enable precise control over cellular behavior and microenvironmental cues through mechanical, electrical, or magnetic stimulation. In this context, organoids, 3D miniaturized organs produced in vitro, have emerged as particularly promising tools due to their capability to replicate key structural and functional features of human organs.^[^ ^7^, ^8^, ^9^ ^]^ This capability has spurred their integration into biohybrid robots, enabling more precise modeling of human systems. Organoids are typically derived from human‐derived induced pluripotent stem cells (iPSCs), offering high accuracy for human‐specific disease research and drug efficacy assays.^[^ ^10^, ^11^ ^]^ When employedd for drug screening, organoid‐based biohybrid robots can deliver more reliable and reproducible results compared to conventional methodologies. Moreover, similar to organ‐on‐a‐chip systems used extensively in biomedical research, biohybrid robots can be immobilized on polymer substrates to create biohybrid robots‐on‐a‐chip.^[^ ^12^, ^13^, ^14^ ^]^ This configuration enhances reproducibility and facilitates easier analysis.

For example, our group previously developed a biohybrid robot‐on‐a‐chip modeled on the human motor system by integrating a brain organoid, multi‐motor neuron spheroids, and muscle bundles.^[^ ^15^ ^]^ This system was successfully employed to evaluate the effects of drugs related to neurodegenerative diseases. By integrating various types of 3D biological tissues, such as brain organoids for signal generation, multi‐motor neuron spheroids for signal transmission, and muscle bundles for movement, onto a polymer chip, the developed biohybrid robot‐on‐a‐chip exhibited enhanced human‐like functionality compared to traditional cell‐based chips. This platform facilitated the analysis of the effects of chemicals on normal brain organoids and the evaluation of drug responses in neurodegenerative disease model brain organoids.

**Figure 1:**
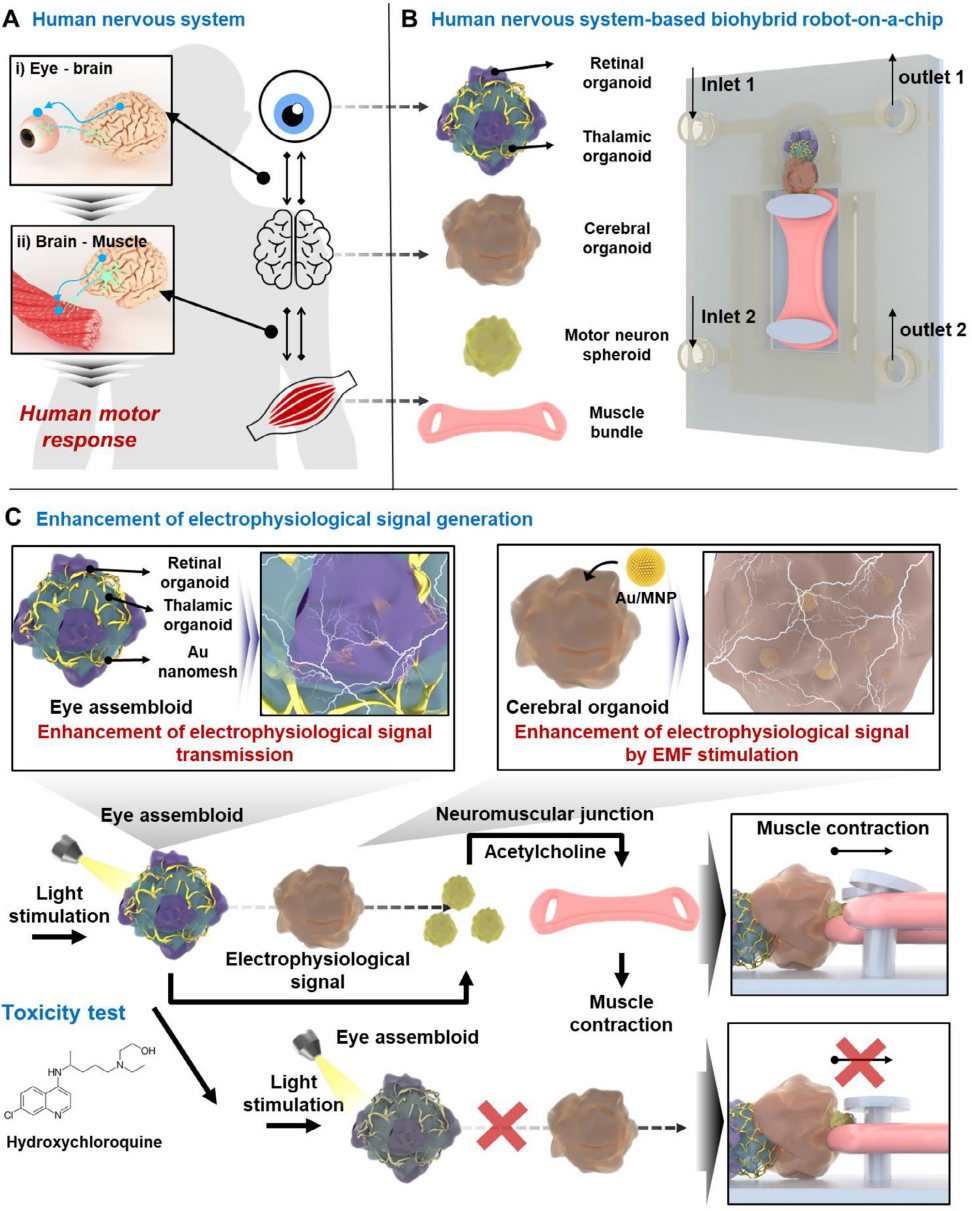
Schematic illustration of the human nervous system‐based biohybrid robot‐on‐a‐chip. A) Schematic images of the human nervous system. B) Schematic representation of the human nervous system‐based biohybrid robot‐on‐a‐chip composed of the eye assembloid, cerebral organoid, motor neuron spheroid, and muscle bundle on the polymer substrate. C) Schematic diagram representing the enhancement of the electrophysiological signal by nanomaterials.

Despite these advances, further research is needed to develop biohybrid robots‐on‐a‐chip that better replicate the complexity of human systems.^[^ ^16^, ^17^ ^]^ Current models often rely on responses to chemical stimuli to mimic motor system functions, which involve the neural and neuromuscular systems. However, human behavior is fundamentally driven by sensory responses, wherein external stimuli are processed by sensory organs and converted into corresponding motor actions (**Figure** **1**A). Sensory organs, such as the eye—which accounts for ≈70% of human sensory input—are crucial for guiding behavior based on external stimuli.^[^ ^18^, ^19^ ^]^ Therefore, to create more human‐like biohybrid robots‐on‐a‐chip, it is essential to incorporate sensory organoids capable of receiving and processing external stimuli.

Retinal organoids, which replicate the retina's function of detecting light and converting it into electrophysiological signals, are promising candidates for enhancing the sensory capabilities of biohybrid robots‐on‐a‐chip.^[^ ^20^, ^21^, ^22^ ^]^ Retinal organoids are composed of neurons capable of both generating and transmitting signals to other biological tissues. Furthermore, their derivation from human iPSCs makes them more stable and adaptable for patient‐specific disease modeling than traditional approaches like optogenetics. However, several challenges must be addressed to effectively integrate retinal organoids into biohybrid robots.^[^ ^23^, ^24^, ^25^ ^]^ The electrophysiological signals generated by retinal organoids are often too weak to stimulate other biological tissues, and connectivity between tissues in vitro is generally poor. To overcome these limitations, nanostructures can be introduced to enhance key properties of biological tissues, including nerve growth, neural plasticity, neurogenesis, and muscle movement.

In this study, we developed a human nervous system‐based biohybrid robot with an eye function as a sensing system in addition to brain/neural network/muscle functions for the first time (Figure 1B). To address the challenges of weak signal generation and poor tissue connectivity, we engineered biological tissues with specific nanostructures (Figure 1C). First, we produced an eye assembloid with enhanced neural network formation and electrophysiological signal transmission functions by attaching retinal organoids to a thalamic organoid wrapped in an Au nanomesh structure. Second, we developed a cerebral organoid with enhanced neurogenesis by introducing Au/magnetic nanoparticles (Au/MNPs). By interfacing the eye assembloid and cerebral organoid with motor neurons and muscle bundles and placing them on a chip, we developed a human nervous system‐based biohybrid robot‐on‐a‐chip. Upon light stimulation, the eye assembloid generated electrophysiological signals, which were transmitted through the cerebral organoid and motor neuron spheroid, inducing muscle movement. Furthermore, treatment with hydroxychloroquine (HCQ), a retinal‐specific toxicant, reduced muscle movement under the same light stimulation intensity, demonstrating the system's sensory function and potential for toxicity screening. The biohybrid robot‐on‐a‐chip with sensing function developed in this study provides a novel platform for future research and drug screening, particularly for complex diseases involving sensory, neural, and muscular systems.

## 2 Results and Discussion

### 2.1 Generation of Retinal and Thalamic Organoids

Retinal and thalamic organoids were generated through the differentiation of iPSCs (Figure S1A, Supporting Information).^[^ ^26^, ^27^ ^]^ Retinal organoid differentiation involved 16 days of neural induction, followed by ≈30 days of induction of retinal formation. This process entailed manually dissecting optic cup segments, which were then differentiated into retinal organoids (Figure 1B). After continued differentiation, retinal organoid development was completed in ≈120 days. The retinal organoids were cultured continuously to promote cell maturation and ensure stable electrophysiological signal production in response to light stimulation. The fully matured 7‐month‐old retinal organoids were used for subsequent experiments.

**Figure 2:**
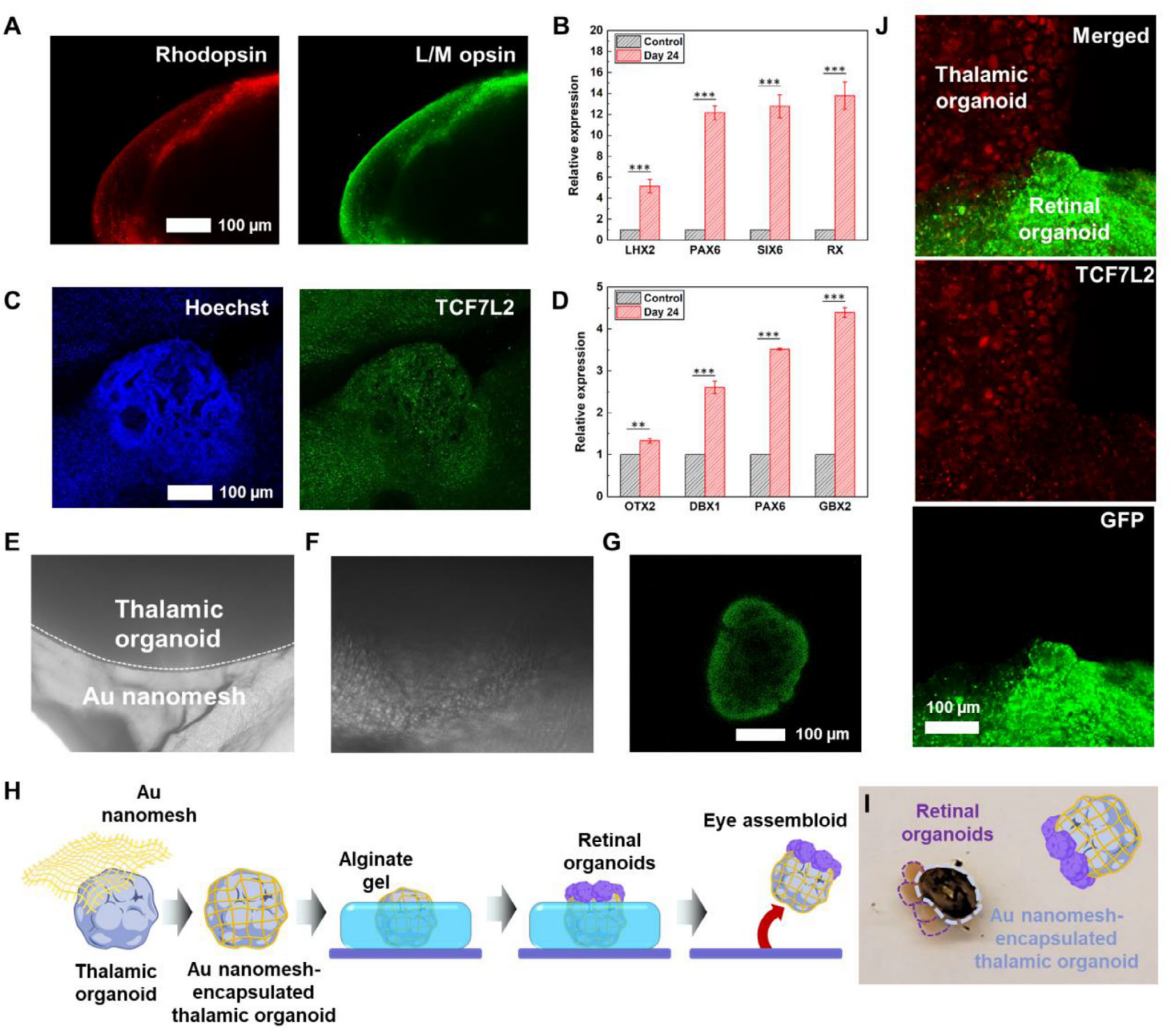
Characterization of retinal/thalamic assembloid. A) Immunostaining images of representative marker expression at day 210 of the retinal organoid. B) qPCR analysis of retinal organoids with LHX2, PAX6, SIX6, and RX genes. ^*^ *p* ≤ 0.05 and ^**^ *p* ≤ 0.01. Error bars show the standard error of the mean of four measurements. C) Immunostaining images of representative marker expression at day 60 of the thalamic organoid. D) qPCR analysis of thalamic organoids with OTX2, PAX6, GBX2, and DBX1 genes. ^*^ *p* ≤ 0.05 and ^**^ *p* ≤ 0.01. Error bars show the standard error of the mean of four measurements. E, F), Optical images of Au nanomesh‐encapsulated thalamic organoid cultured for 30 days. G) Fluorescence image of thalamic organoid incubated with fluorescent‐tagged BSA. H) Schematic illustrations of the fabrication process of the eye assembloid. I) Snapshot image of the eye assembloid. J) Immunostaining images of the eye assembloid.

Immunostaining revealed that optic nerve cells, which are crucial for generating electrophysiological signals in response to light, were formed on the surface of the retinal organoids. Specifically, the identification of L/M opsin proteins, along with rhodopsin proteins, indicated the presence of optic nerve cells, including ganglion and cone cells, which generate electrophysiological signals in response to light contrast or varying wavelengths (**Figure** **2**A).^[^ ^28^ ^]^ To further analyze the retinal organoids, gene expression was evaluated. The expression levels of four key genes involved in the early stages of neuronal differentiation and retinal formation (LHX2, PAX6, SIX6, and RX) were analyzed using quantitative polymerase chain reaction (qPCR). These genes showed a significant increase in expression compared to their levels prior to differentiation (Figure 2B; Table S1, Supporting Information).

Next, we analyzed whether the retinal organoids could generate electrophysiological signals in response to light. For this, retinal organoids were placed on a multielectrode array (MEA) plate with 16 individual electrodes. Light stimulation was provided via a flexible optic cable connected to a light‐emitting diode (LED) illuminator positioned 15 cm away from the MEA plate in a darkroom. White light containing four different wavelengths was irradiated onto the retinal organoids at irregular intervals to ensure that the retinal organoids generated electrophysiological signals only in response to light stimulation. The light was briefly applied for one second and then turned off. As shown in Figure S2 (Supporting Information), the retinal organoids immediately generated electrophysiological signals upon light exposure, with most electrodes recording simultaneous signals, confirming that the retinal organoids could transmit electrical signals, similar to the human eye.

Thalamic organoids were generated through neuroinduction, thalamic patterning, and neural differentiation processes (Figure S1C, Supporting Information). The thalamic organoids were cultured for ≈21 days and then further matured for an additional culture period of over 2 months, similar to the maturation of retinal organoids. Immunostaining revealed a well‐formed rosette structure characteristic of thalamic organoids (Figure 2C). Additionally, gene expression analysis via qPCR confirmed the successful production of thalamic organoids, with all four genes (OTX2, DBX1, PAX6, and GBX2) showing increased expression levels compared to their pre‐differentiation levels (Figure 2D; Table S1, Supporting Information). In summary, both retinal and thalamic organoids were successfully generated.

### 2.2 Fabrication of Au Nanomesh for Encapsulating Thalamic Organoids

**Figure 3:**
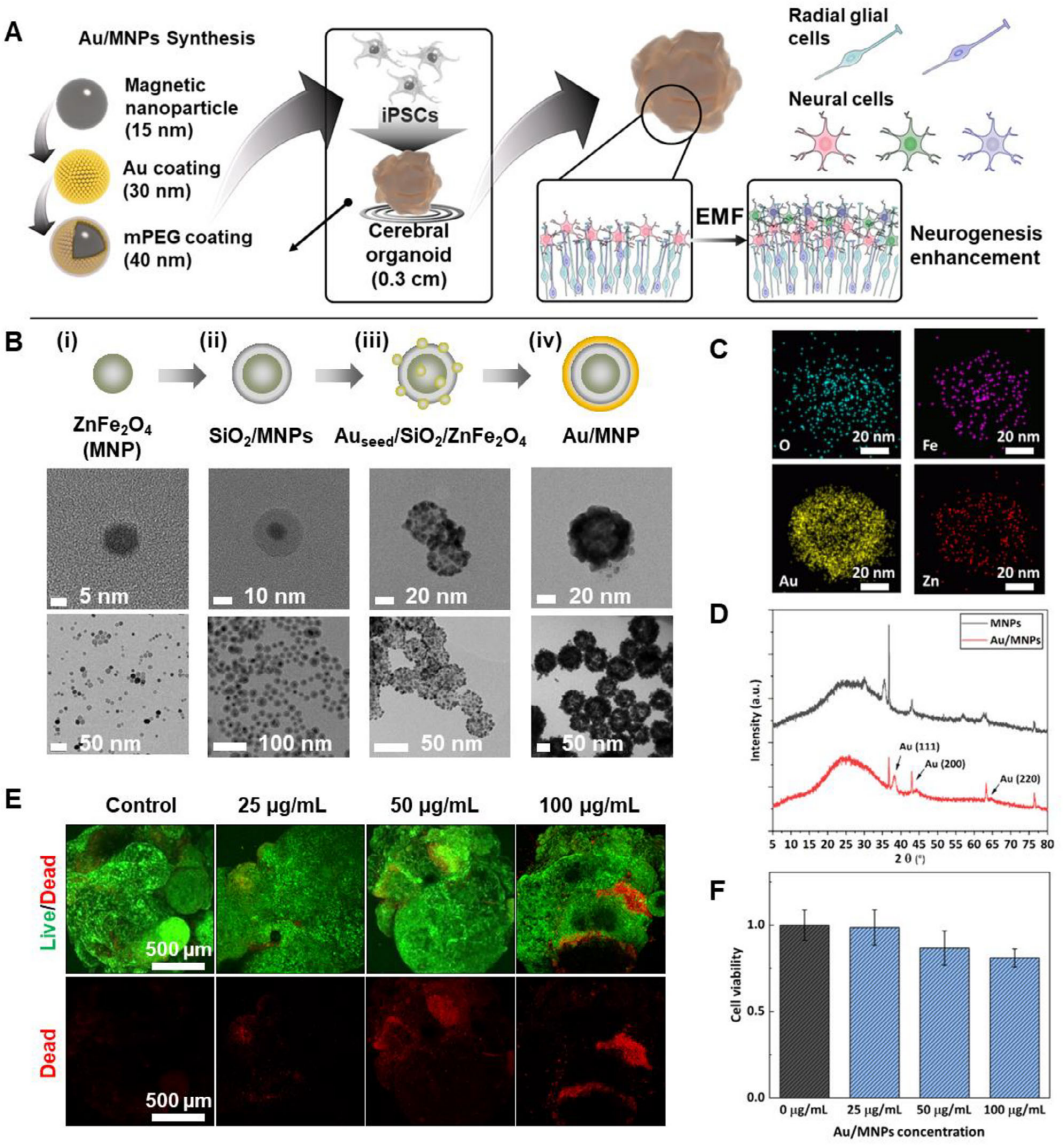
Synthesis of Au/MNPs. A) Schematic illustrations of the effect of Au/MNP on cerebral organoid. B) Synthesis of Au/MNPs included four steps: i) ZnFe_2_O_4_ nanoparticles (MNPs), ii) SiO_2_/MNPs, iii) Au seed/SiO_2_/MNPs, and iv) Au/MNPs. C) EDS mapping of Au/MNP. D) X‐ray crystallography (XRD) analysis of MNPs and Au/MNPs. E) Live and dead images and F) 3D cell viability assay of Au/MNPs on cerebral organoid.

To improve the connection efficiency between thalamic and retinal organoids, Au nanomesh was fabricated and applied to encapsulate the thalamic organoids. The integration of Au nanomesh was strategically selected to enhance electrical coupling between the retinal and thalamic organoids due to its superior biocompatibility, flexibility, and ability to form conformal contact with 3D tissues compared to conventional planar electrodes.^[^ ^29^ ^]^ This is particularly advantageous when working with organoids, whose shapes and sizes are highly variable and not standardized, as the nanomesh can adapt to irregular surfaces and maintain consistent contact without damaging the tissue.^[^ ^30^ ^]^ Alternative materials, such as ITO or carbon‐based nanomaterials, were considered but lacked the mechanical flexibility or introduced higher cytotoxicity risks, which could impair long‐term organoid viability.^[^ ^31^, ^32^ ^]^ The Au nanomesh was fabricated using the electrospinning method (**Figure** **3**A). The electrospun PVA nanomesh displayed randomly arranged nanofibers with uniform thickness (450 nm) and a smooth surface (Figure S3B,C, Supporting Information). After Au deposition, the surface roughness increased significantly compared to PVA alone (Figure 3D,E). An enlarged FE‐SEM image of the Au nanomesh, following PVA dissolution, revealed nanofibers with a uniform thickness of 570 nm, slightly larger than the PVA nanofibers.

The fabricated Au nanomesh was then used to encapsulate the thalamic organoids, and the Au nanomesh‐encapsulated thalamic organoids were cultured for 7 days. The Au nanomesh‐encapsulated thalamic organoids retained their morphology without structural collapse for over 30 days following the introduction of the Au nanomesh (Figure 2E,F). To assess the functionality of the nanomesh, fluorescent‐tagged bovine serum albumin (BSA) was applied to the Au nanomesh‐encapsulated thalamic organoids. ≈30 min post‐BSA treatment, the BSA molecules diffused efficiently through the Au nanomesh into the thalamic organoids, as confirmed by fluorescence imaging (Figure 2G). To verify the biocompatibility of the Au nanomesh introduced onto the thalamic organoid, it was necessary to assess whether their encapsulation affected cellular apoptosis or inflammatory responses. Therefore, additional experiments were conducted to evaluate apoptosis and immune activation, demonstrating that the Au nanomesh exhibited high biocompatibility, thus making it suitable for encapsulation onto the thalamic organoid without compromising their development or functionality. As shown in Figure S4A (Supporting Information), the Au nanomesh‐encapsulated thalamic organoids exhibited Caspase‐3 activation to a level comparable to that of the control group (Thalamic organoid). Furthermore, to further evaluate inflammatory responses, we quantified TNF‐α, a key pro‐inflammatory cytokine commonly used as a biomarker for immune activation and tissue inflammatory status. TNF‐α levels were measured to assess whether Au nanomesh encapsulation induced abnormal immune responses to the thalamic organoids. The quantitative analysis revealed that the expression levels of TNF‐α in the Au nanomesh‐encapsulated thalamic organoids (60.1 ± 1.28 pg mL^−1^) were comparable to those observed in the control group (without Au nanomesh) (24.1±3.06 pg mL^−1^), indicating that Au nanomesh encapsulation did not induce significant inflammatory activation (Figure S4B, Supporting Information). In addition, there were reports that a TNF‐α concentration below ≈250 pg mL^−1^ does not significantly impact the functionality of the organoid, which is consistent with our results.^[^ ^33^, ^34^ ^]^ This result confirmed that, despite the Au nanomesh completely encapsulating the thalamic organoids, nutrients and wastes could still pass through the mesh gaps, as hypothesized. In addition to this, the results demonstrated minimal apoptotic activity and low levels of inflammatory marker expression, indicating that the Au nanomesh exhibited excellent biocompatibility and was well integrated with the thalamic organoid.

Finally, electrophysiological signals generated by the thalamic organoids were measured both with and without Au nanomesh encapsulation. In both cases, the thalamic organoids continuously generated electrophysiological signals (Figure S5, Supporting Information). Even with the Au nanomesh covering the surface, there were no obstructions to measuring the electrophysiological signals due to the excellent electrical conductivity of Au.

### 2.3 Fabrication of the Eye Assembloid

The eye assembloid was fabricated by attaching multiple retinal organoids to the Au nanomesh‐encapsulated thalamic organoid using an alginate‐based sacrificial hydrogel for precise placement (Figure 2H,I). Fluorescence imaging confirmed the attachment of retinal organoids to the Au nanomesh‐encapsulated thalamic organoid. GFP fluorescence signals from GFP‐expressing retinal organoids were observed along the boundaries of the Au nanomesh‐encapsulated thalamic organoid, indicating neuronal projections from the retinal organoids. Notably, TCF7L2‐expressing neurons from the Au nanomesh‐encapsulated thalamic organoid (shown in red) were found to extend across the entire surface of the retinal organoids (shown in green) (Figure 2J). To verify the improvement in connection efficiency enabled by the Au nanomesh, an eye assembloid was fabricated without the Au nanomesh. Compared to the nanomesh‐enabled fabrication, significantly fewer cell projections were observed in assembloids without the Au nanomesh (Figure S6A, Supporting Information).

Next, the electrophysiological signals generated by the fabricated eye assembloid were measured to evaluate the connectivity between retinal and thalamic organoids. To test the transmission of light‐induced signals, the Au nanomesh‐encapsulated thalamic organoid was selectively contacted with MEA electrodes while light was applied to the retinal organoids. Upon light stimulation, continuous electrophysiological signals were generated from the Au nanomesh‐encapsulated thalamic organoid (Figure S6B, Supporting Information), confirming effective connectivity between the retinal and the Au nanomesh‐encapsulated thalamic organoids. Next, to assess the role of the Au nanomesh in enhancing this connection, electrophysiological signals from assembloids without the nanomesh were also measured. Although light‐induced signals were generated, the signal intensity in assembloids with the Au nanomesh was ≈1.7 times higher than that without the Au nanomesh (Figure S6C, Supporting Information). This demonstrated that the Au nanomesh significantly improved signal transmission efficiency. Thus, the eye assembloid, with its enhanced light‐responsive electrophysiological signal transmission, holds potential as a visual sensor in biohybrid robots, functioning as an artificial eye.

### 2.4 Synthesis of Au‐Coated MAGNETIC Nanoparticles (Au/MNPs)

To enhance neurogenesis in cerebral organoids, Au/MNPs were synthesized and incorporated within the cerebral organoid (Figure 3A). The synthesis process of Au/MNPs involved four key steps: i) synthesis of ZnFe_2_O_4_ nanoparticles (MNPs), ii) encapsulation of MNPs with a silica (SiO_2_) layer (forming SiO_2_/MNPs), iii) attachment of Au seeds onto the SiO_2_/MNPs (forming Au_seed_/SiO_2_/MNPs), and iv) growth of the Au layer on the Au_seed_/SiO₂/MNPs to form the final Au/MNPs (Figure 3B). The transmission electron microscopy (TEM) images for each step are shown in Figure 3B (i–iv). We began by synthesizing monodispersed Zn‐doped MNPs through the thermal decomposition of iron (III) acetylacetonate and zinc (II) chloride in trioctylamine buffer using 1,2‐hexadecanediol, oleylamine, and oleic acid. The resulting MNPs had an average diameter of 8.0 ± 1.06 nm (Figure 3B(i)). The MNPs were then encapsulated in a silica shell using a standard water‐in‐oil microemulsion method. Upon adding tetraethyl orthosilicate (TEOS) to the microemulsion, hydrolysis occurred at the water‐oil interface, followed by ligand exchange with the chemically absorbed Igepal CO‐520 on the surface of the MNPs. This process facilitated the migration of MNPs into the aqueous phase, where hydrolyzed TEOS formed a silica shell ≈10 nm thick. The size of the prepared SiO_2_/MNPs increased to 24.52 ± 2.65 nm, compared to the initial size of the MNPs (Figure 3B(ii)). Next, the surface of SiO_2_/MNPs was modified with (3‐aminopropyl)triethoxysilane (APTES), which facilitated the attachment of Au seeds via electrostatic interactions. The diameter of the resulting Auseed/SiO_2_/MNPs increased to 31.01 ± 2.53 nm due to the addition of 5 nm‐sized Au seeds (Figure 3B(iii)). Finally, the prepared Au_seed_/SiO_2_/MNP was reacted with an Au growth solution to generate the Au/MNPs (Figure 3B(iv)). The elemental composition of the synthesized Au/MNPs was confirmed using energy‐dispersive X‐ray (EDS) mapping, which revealed the presence of Au, Fe, and Zn components (Figure 3C). Additionally, UV–vis spectrophotometry confirmed the presence of Au on the surface of the Au/MNPs, with an absorption maximum observed at 565 nm (Figure S7A, Supporting Information). Additionally, X‐ray diffraction (XRD) analysis was performed at a scanning rate of 10°/min over a 2θ range of 5° to 80°. The XRD patterns exhibited characteristic peaks at (111), (200), and (220), confirming the successful Au coating of the SiO_2_/MNPs (Figure 3D).To improve the biocompatibility of Au/MNPs for incorporation into cerebral organoids, the surface of the Au/MNPs was further modified with methoxy‐polyethylene glycol thiol (mPEG‐SH). The surface modification was confirmed via zeta‐potential analysis (Figure S7B, Supporting Information), which showed a shift from −25.05 ± 0.92 mV to −18.81 ± 0.58 mV, indicating the successful attachment of mPEG, which introduced a positive charge to the nanoparticles.

### 2.5 Generation of Au/MNP‐Incorporated Cerebral Organoids to Promote Neurogenesis

**Figure 4:**
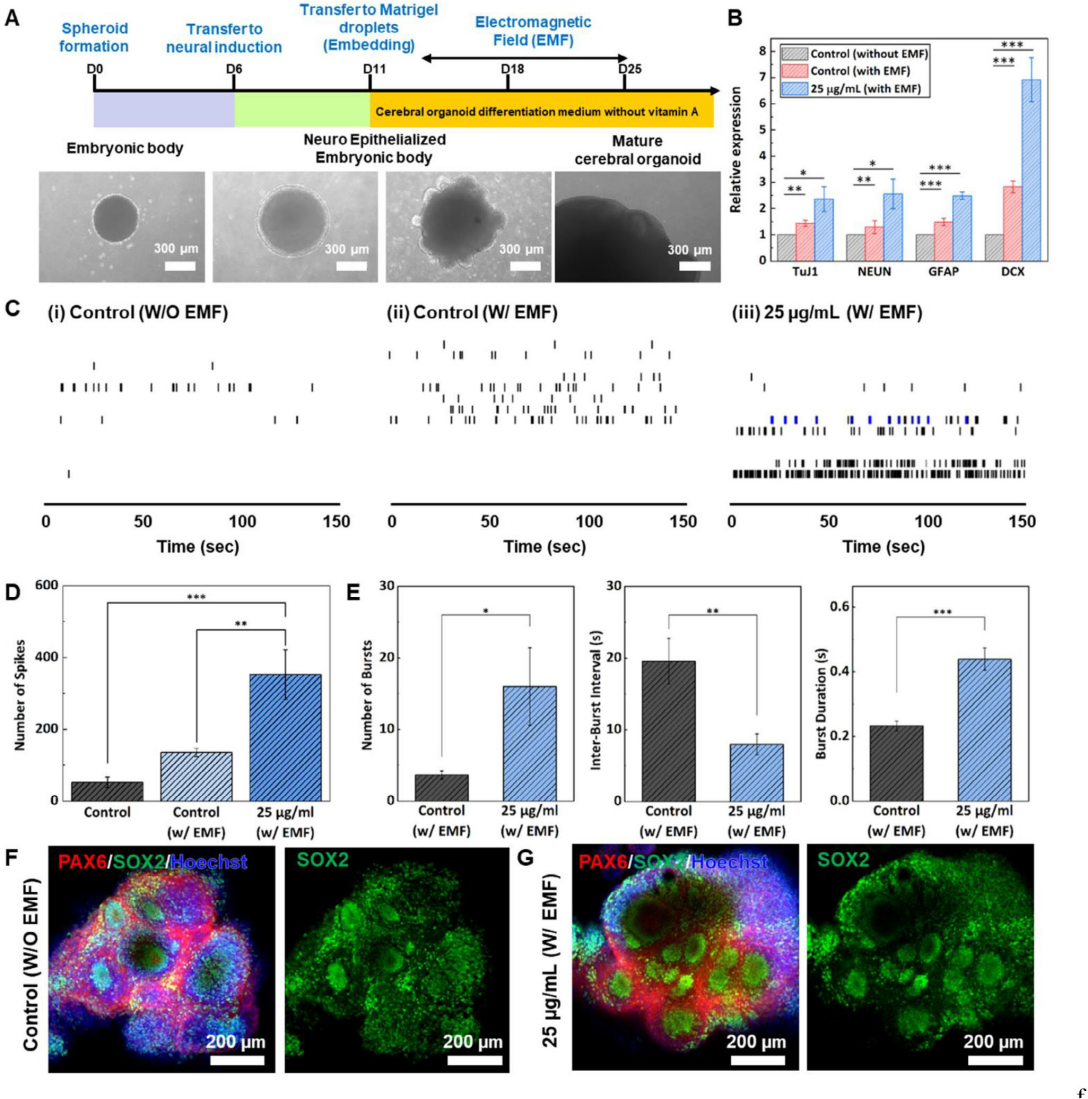
Generation of Au/MNPs‐incorporated cerebral organoid. A) The generation process of Au/MNPs‐incorporated cerebral organoid. B) Characterization of Au/MNPs‐incorporated cerebral organoid. Normalized expression levels of TuJ1, NEUN, GFAP, and DCX. ^*^ *P* < 0.05, ^**^ *P* < 0.01, and ^***^ *P* < 0.001. Error bars correspond to the standard error of the mean from three measurements. C) Spike raster plot, and D) number of spikes of cerebral organoid according to EMF stimulation and Au/MNPs. ^**^ *P* < 0.01, and ^***^ *P* < 0.001. Data are presented as mean ± SEM, and statistical analyses were performed using a paired comparisons test (n = 3 independent organoids per sample recorded and 2 independent experiments performed). E) Number of bursts, inter‐burst interval, and burst duration of Au/MNPs‐incorporated cerebral organoid. ^*^ *P* < 0.05, ^**^ *P* < 0.01, and ^***^ *P* < 0.001. Data are presented as mean ± SEM, and statistical analyses were performed using a paired comparisons test (n = 3 independent organoids per sample recorded and 2 independent experiments performed). Immunostaining images of representative marker expression at day 30 of cerebral organoid F) without Au/MNPs, EMF stimulation, and G) with Au/MNPs, EMF stimulation.

To explore the potential of Au/MNPs in promoting neurogenesis within cerebral organoids, Au/MNPs were incorporated during the cerebral organoid generation process. First, the biocompatibility of Au/MNPs with cerebral organoids was evaluated using 3D cell viability and live/dead assays. In the results of the live/dead assay (Figure 3E), green indicates live cells and red indicates dead cells. Four final concentrations of Au/MNPs were tested: 0 (control), 25, 50, and 100 µg mL^−1^. The cerebral organoids, evaluated 7 days after embedding, showed varying results depending on the Au/MNP concentration. As the Au/MNP concentration increased, the number of dead cells also increased. Notably, a rapid increase in dead cells was observed when the concentration of Au/MNPs exceeded 50 µg mL^−1^. However, the survival rate of cerebral organoids incorporating 25 µg mL^−1^ Au/MNPs remained high at 98.61% after 10 days, compared to the control, indicating the high biocompatibility of Au/MNPs (Figure 3F). Furthermore, we conducted an additional live/dead assay to provide detailed data for the biocompatibility of the Au/MNP. To verify the biocompatibility of the Au/MNP, we directly added the Au/MNPs to the embryoid bodies (EBs). After 7 days of Au/MNPs treatment, the EBs with the 50 µg mL^−1^ concentration of treated nanoparticles only indicated a high density of dead cells compared with the EBs below the 50 µg mL^−1^ concentration (Figure S8, Supporting Information). Building on this promising result, a solenoid coil was designed and simulated to generate an electromagnetic field (EMF) of 0.2 mT (Figure S9, Supporting Information). In particular, EMF stimulation was employed to promote neurogenesis and neuronal network maturation non‐invasively within cerebral organoids. Compared to conventional chemical induction or direct electrical stimulation, EMF stimulation offers the advantage of uniform field penetration, reduced local heating, and minimal physical contact, making it highly compatible with complex 3D organoid systems. As shown in **Figure** **4**A, the cerebral organoid was initially generated through the formation of EBs, followed by the neuroepithelization of the EBs.^[^ ^35^ ^]^ The outer tissue of the EBs became more distinguishable, exhibiting a brighter appearance, on day 6 after EB generation. Subsequently, neuroectodermal tissue began to form around the surface of the early EB. By day 11, the neuroectodermal tissue was embedded into Matrigel to support its growth into neuroepithelial tissue. To enhance neurogenesis during this process, Au/MNPs were incorporated into the Matrigel. After 7 days of embedding, the organoid was subjected to 1 h of electromagnetic field (EMF) stimulation (0.2 mT, 60 Hz) daily for 7 days. Neurogenesis in the cerebral organoids was confirmed by performing a qPCR analysis of neurogenesis‐related genes, including TuJ1, NEUN, GFAP, and DCX (Figure 4B; Table S1, Supporting Information). The expression levels of TuJ1, NEUN, GFAP, and DCX were upregulated by 2.36, 2.56, 2.49, and 6.92 times, respectively, compared to the control group without Au/MNPs and EMF stimulation. These findings demonstrate that the combined effect of Au/MNPs and EMF stimulation significantly enhanced neurogenesis in the cerebral organoids, potentially improving their electrophysiological activity.

### 2.6 Confirmation of Electrophysiological Signal Enhancement and Neurogenesis by Au/MNPs and EMF

To assess the impact of Au/MNPs and EMF on neurogenesis and electrophysiological activity, cerebral organoids from three experimental groups were tested on day 60 (Figure 4C): i) control (without EMF), ii) control (with EMF), and iii) 25 µg mL^−1^ Au/MNPs (with EMF). These organoids were loaded onto a 64‐electrode MEA system for electrophysiological signal analysis. The surface of the MEA system was coated with 0.5% poly(ethyleneimine) (PEI) to facilitate the attachment of the organoids. After attachment, electrophysiological signals were measured following 5 days of incubation. The control group without EMF showed the lowest electrophysiological signal activity. However, when EMF stimulation was applied to this group, a significant increase in signal activity was observed. In the Au/MNPs‐incorporated groups, both Au/MNPs and EMF stimulation significantly enhanced the electrophysiological signals. Specifically, the number of spikes in the Au/MNP‐incorporated cerebral organoids increased by 6.75 times compared to the control group without Au/MNPs and EMF stimulation (Figure 4D). Additionally, the number of bursts increased from 3.67 ± 0.56 to 16 ± 5.43 in the Au/MNPs incorporated cerebral organoids, and the inter‐burst interval was reduced from 19.57 ± 3.17 s to 7.98 ± 1.43 s (Figure 4E). The duration of the bursts also improved, increasing from 0.23 ± 0.014 to 0.44 ± 0.034 s compared to the control. Furthermore, the enhancement of neurogenesis not only led to an increased frequency of electrophysiological signals but also resulted in a notable amplification of the signal strength. This amplification is likely attributed to the accelerated neuronal maturation and improved functional connectivity among neurons, both of which contribute to more synchronized and robust action potential generation within the cerebral organoids. As shown in Figure S10 (Supporting Information) the electrophysiological signal in the Au/MNP‐incorporated cerebral organoid (19.0 ± 0.53 µV) indicated 1.49 times higher action potential compared to the control group (12.7 ± 0.35 µV). These results indicated that the enhancement of neurogenesis not only led to an increased frequency of electrophysiological signals but also resulted in a notable amplification of the signal strength. This amplification is attributed to the accelerated neuronal maturation and improved functional connectivity among neurons, both of which contribute to more synchronized and robust action potential generation within the cerebral organoids.

These electrophysiological enhancements were accompanied by cellular changes, as confirmed by immunostaining for progenitor cell markers. Specifically, immunostaining for SOX2 and PAX6 on day 30 revealed a higher density of radial glial cells along the ventricular zone (VZ), while neural markers TuJ1 and DCX were also enhanced, indicating increased neurogenesis (Figure 4F,G). As neurogenesis progressed, the number of cells and the size of the organoid increased. On day 60, the area of the Au/MNPs‐incorporated cerebral organoids expanded by 1.65 times, from 3.28 ± 0.55 mm^2^ to 5.41 ± 1.15 mm^2^, compared to the control group without Au/MNPs and EMF stimulation (Figure S11, Supporting Information). These results demonstrate that the combination of Au/MNPs and EMF stimulation significantly enhances both the electrophysiological activity and neurogenesis within cerebral organoids.

### 2.7 Verification of Functional Connectivity between Organoids, Motor Neuron Spheroids, and Muscle Bundles

**Figure 5:**
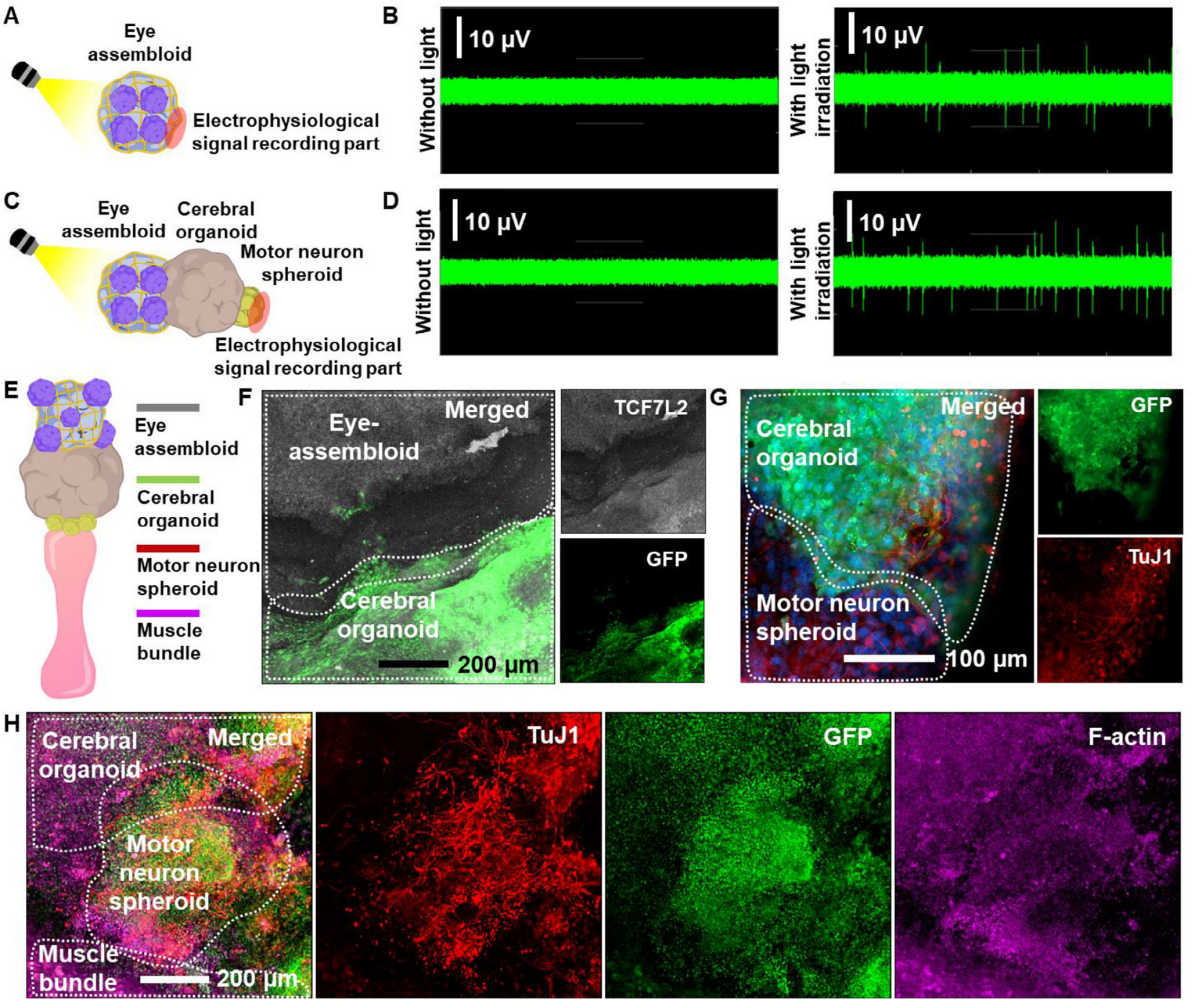
Conjugation of eye assembloid‐cerebral organoids‐MNSs with muscle bundle. A) Schematic illustrations and B) electrophysiological signals of the eye assembloid. C) Schematic illustrations and D) eye assembloid‐cerebral organoid‐motor neuron spheroid connections. E) Schematic illustrations of connected cell structures and F) optical images of connected cerebral organoid, motor neuron spheroid, and muscle bundle. Immunostaining images of representative marker expression of the G) eye assembloid and H) cerebral organoid, motor neuron spheroids, and muscle bundle.

To verify functional connectivity, electrophysiological signals were measured across various organoid structures (**Figure** **5**). For the eye assembloid, light stimulation was applied to the retinal organoids, and the resulting electrophysiological signals generated by the thalamic organoids were recorded (Figure 5A). Compared to the control (without light), a significantly larger number of action potentials were continuously generated during light application (Figure 5B). Next, the transmission of electrophysiological signals from the eye assembloid to the motor neuron spheroid through the cerebral organoid was analyzed (Figure 5C). For this, the eye assembloid was attached to the cerebral organoid with a motor neuron spheroid. Then, after co‐culturing the eye assembloid, cerebral organoid, and motor neuron spheroid, light stimulation was applied to the eye assembloid to analyze the electrophysiological signals transmitted to the motor neuron spheroid. As with the eye assembloid, a significantly higher number of action potentials was generated during light application compared to the control (Figure 5D). These results confirm that the eye assembloid, cerebral organoid, and motor neuron spheroid are functionally connected through an intercellular neural network, enabling light‐induced electrophysiological signal generation and transmission.

To further validate these connections, immunostaining was performed (Figure 5E, F; Figure S12, Supporting Information). First, immunostaining for TCF7L2, a thalamus‐specific biomarker, was conducted 3 days after conjugation of the eye assembloid with the cerebral organoid (Figure 5G). Additionally, cerebral organoids generated using GFP‐expressing iPSCs were used to confirm the formation of intercellular networks. The results confirmed that the thalamic organoid and cerebral organoid were successfully connected (Figure 5G), even though they did not directly touch. Instead, a network of cells extending from both organoids was observed, indicated by overlapping regions of gene expression. Next, immunostaining was performed on the connected cerebral organoid‐motor neuron spheroid‐muscle bundle system to confirm the connection. Similar to the previous analysis, GFP‐expressing cells in the cerebral organoid were used to verify the connection. As shown in Figure 5H, GFP expression in the cerebral organoid overlapped with the position of the motor neuron spheroid, while the neuron marker TuJ1 was detected next to F‐actin. These results demonstrate that each 3D cell structure—eye assembloid, cerebral organoid, motor neuron spheroid, and muscle bundle—was successfully connected.

### 2.8 Development and Functional Validation of a Human Nervous System‐Based Biohybrid Robot‐On‐A‐Chip

**Figure 6:**
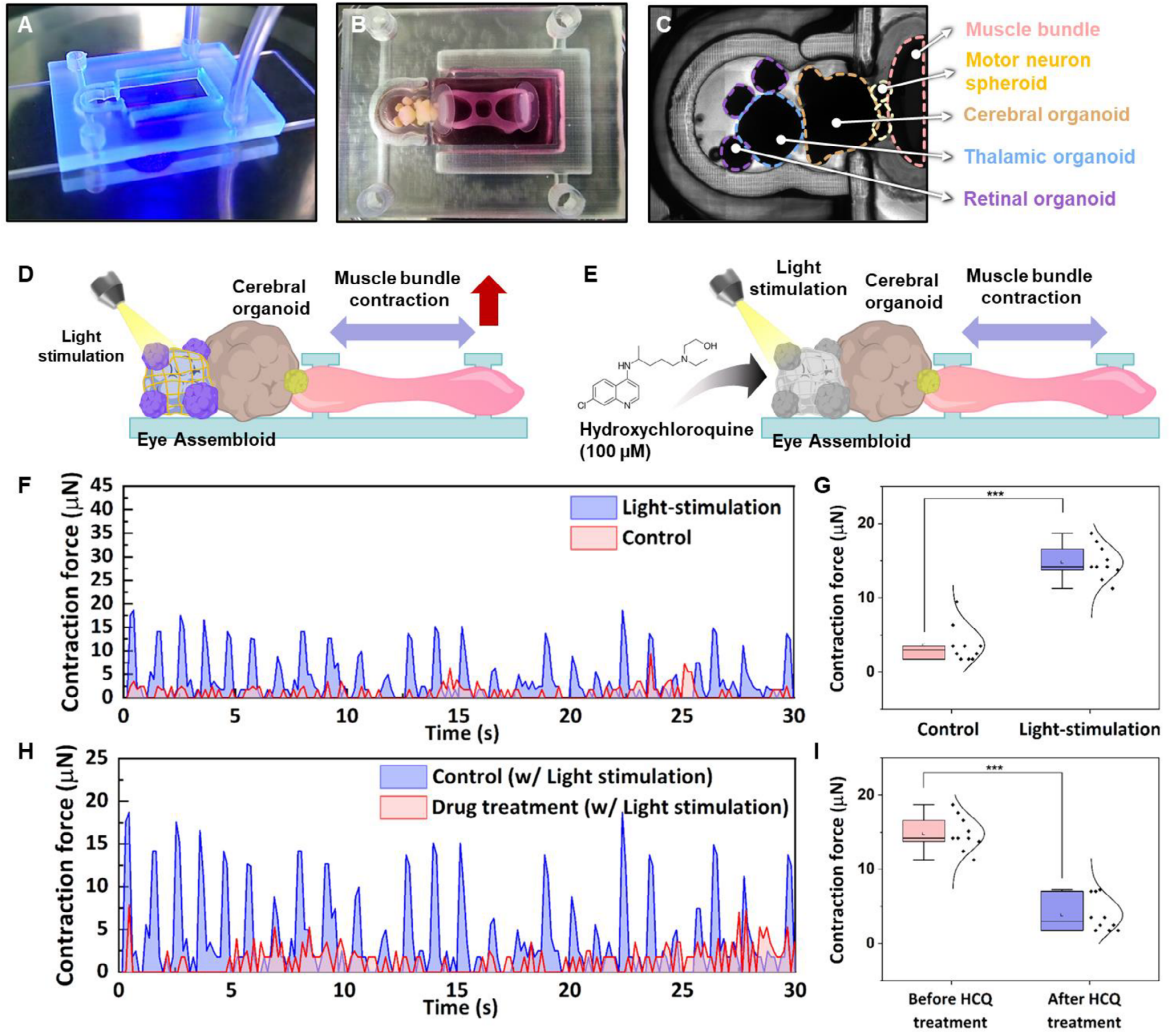
Generation and confirmation of human nervous system‐based biohybrid robot‐on‐a‐chip. Optical image of A) 3D printed chip and B, C) human nervous system‐based biohybrid robot‐on‐a‐chip. D) Schematic images of the muscle bundle contraction of human nervous system‐based biohybrid robot‐on‐a‐chip, and E) hydroxychloroquine effect evaluation by muscle bundle contraction of human nervous system‐based biohybrid robot‐on‐a‐chip. F, G) Light stimulation induced‐contraction force of the muscle bundle in the human nervous system‐based biohybrid robot‐on‐a‐chip. H, I) Hydroxychloroquine‐induced contraction force of the muscle bundle in the human nervous system‐based biohybrid robot‐on‐a‐chip.

A 3D printed chip was designed Using 3D printing technology to fabricate the human nervous system‐based biohybrid robot‐on‐a‐chip (Figure 6A; Figure S13, Supporting Information). The chip design featured two distinct channels: Channel 1 for the eye assembloid and cerebral organoid (blue) and Channel 2 for the motor neuron spheroid and muscle bundle (red). The components were co‐cultured for ≈2 weeks to confirm their response to light stimulation.

First, the differentiated eye assembloid and cerebral organoid were positioned in Channel 1 of the chip, and then the muscle bundle was transferred and connected to the motor neuron spheroid in Channel 2 using Matrigel. After co‐culturing the eye assembloid and cerebral organoid in the maturation medium for Channel 1, and the motor neuron spheroid and muscle bundle in the maturation medium for Channel 2, the human nervous system‐based biohybrid robot‐on‐a‐chip was fully assembled, and its connectivity was confirmed through optical imaging (Figure 6B). After 1 week of co‐culture, each organoid and 3D structure demonstrated successful integration within the chip (Figure 6C).

To test functional connectivity, muscle bundle contraction in response to light stimulation was investigated (Figure 6D). Electrophysiological signals generated in the eye assembloid under light stimulation were transmitted to the muscle bundle through the cerebral organoid and motor neuron spheroid, enhancing muscle contraction (Figure 6F). To quantify muscle bundle movement, we tracked its contraction using a microscope. The contraction was recorded for 30 s both before (control) and after light stimulation (Movie S1, Supporting Information). To measure the contraction force, a polymer substrate with Ecoflex pillars (elastic modulus, E = 0.125 MPa) was used. The distance (δ) the pillar edge moved due to muscle contraction was recorded and used to calculate the contraction. As a result of light‐induced electrophysiological signals, the contraction force of the muscle bundle increased to 14.79 ± 2.28 µN, representing a 4.04‐fold increase in movement compared to the control (Figure 6F,G).

The biohybrid robot‐on‐a‐chip was further validated as a platform for retinal toxicity screening. To assess retinal toxicity, muscle bundle contraction was first evaluated after light stimulation (Figure 6E). The contraction was then analyzed following treatment with 100 µM of HCQ, a compound known to be toxic to photoreceptor cells in retinal organoids.^[^ ^36^ ^]^ After HCQ treatment, the contraction force of the muscle bundle within the human nervous system‐based biohybrid robot‐on‐a‐chip reduced from 14.79 ± 2.28 µN to 3.86 ± 2.33 µN (Figure 6H, I; Movie S2, Supporting Information). This reduction is attributed to HCQ‐induced damage to the photoreceptor cells, which impaired their ability to receive light and transmit electrophysiological signals to the muscle bundle. Furthermore, we successfully assessed the dose‐dependent effects of hydroxychloroquine (HCQ) in the range of 5–100 µM, clearly demonstrating its ability to detect drug‐induced disruptions across the entire sensory‐to‐motor circuit (Figure S14, Supporting Information). These results demonstrate that our system enables functional toxicity evaluation through electrophysiological signal changes. To the best of our knowledge, this is the first platform integrating multiple human organoids, including eye, brain, neural, and muscle tissues, within a single system, allowing sensitive detection of toxic effects reflected in downstream functional impairments. Additionally, the chip‐based modular design of the system not only enables potential scalability for drug screening applications but also offers the possibility of reducing costs in future developments by facilitating efficient customization and reuse of individual organoid modules.

## 3 Conclusion

In this study, we introduced a sensing function to the biohybrid robot‐on‐a‐chip, incorporating an eye/brain/neural network/muscle system composed of eye assembloids, cerebral organoids, motor neuron spheroids, and muscle bundles on a polymer substrate. This novel biohybrid robot‐on‐a‐chip integrates eye assembloids, which convert light into electrophysiological signals, and thalamic organoids that enhance signal transmission. The eye assembloid, composed of retinal and thalamic organoids, was generated to achieve the sensing function. The photoreceptors inside the eye assembloid convert external light stimulation into electrophysiological signals, which are then recognized by cerebral organoids and transmitted to muscle cells via the motor neuron spheroid to trigger muscle cell contraction. The introduction of Au nanomesh in the eye assembloid improved signal transmission from retinal to thalamic organoids, while Au/MNP‐incorporated cerebral organoids enhanced neurogenesis and electrophysiological activity. These innovations enabled light‐induced electrophysiological signals to control muscle bundle contraction, increasing the contraction force by 4.04 times. Additionally, HCQ treatment caused a reduction in contraction force, from 14.79 ± 2.28 µN to 3.86 ± 2.33 µN, indicating the system's responsiveness to toxic stimuli.

These results confirm that the developed biohybrid robot‐on‐a‐chip effectively mimics human‐like muscle contractions in response to light stimulation, providing a promising alternative to animal testing for drug evaluation. This system can be applied to evaluate drugs targeting eye diseases, neurodegenerative diseases, and muscle disorders. However, despite these advancements, our current biohybrid robot‐on‐a‐chip system still presents limitations in fully mimicking the complexity of human physiological environments. In the human body, muscle contraction and neural activity are influenced by systemic interactions among various organs, including metabolic, vascular, respiratory, gastrointestinal, and integumentary systems. These organ systems contribute to homeostasis, drug metabolism, immune responses, and sensory processing, which are critical when evaluating drug effects and modeling disease pathology.

To overcome these limitations and further enhance the physiological relevance of the system, future iterations of the biohybrid robot‐on‐a‐chip could benefit from integrating additional human organoids representing key organ systems. For instance, introducing blood vessel organoids or engineered endothelial networks would enable the modeling of systemic circulation, allowing dynamic delivery of nutrients, oxygen, and pharmacological agents.^[^ ^37^, ^38^ ^]^ This would simulate drug absorption, distribution, and clearance more accurately, providing insights into pharmacokinetics and systemic toxicity that are challenging to evaluate in the current platform. Furthermore, incorporating lung organoids would enable the simulation of gas exchange processes, allowing investigation into how oxygenation status or respiratory dysfunction affects muscle contraction or neural activity.^[^ ^39^ ^]^ Intestinal and liver organoids could be integrated to model first‐pass metabolism, digestive absorption, and gut‐brain axis interactions.^[^ ^40^, ^41^ ^]^ The gut microbiome's role in modulating neural function and systemic inflammation is increasingly recognized, and its inclusion would expand the system's application to neurodegenerative diseases, inflammatory bowel disease, or drug metabolism studies. To achieve dynamic and functional integration of these organ systems, advancements in microfabrication and bioengineering are essential. Incorporating microfluidic perfusion systems would facilitate continuous nutrient and drug delivery, real‐time sampling, and waste removal, supporting long‐term culture stability and functional assays. Organ‐specific perfusable channels could be used to model tissue‐tissue interactions and temporal drug exposure scenarios.

In terms of analytical capabilities, expanding real‐time functional monitoring methods will be critical. Techniques such as calcium imaging, voltage‐sensitive dye recordings, and optogenetic reporters could allow detailed spatiotemporal analysis of electrophysiological responses across the neural‐muscular axis. Simultaneously, non‐invasive imaging modalities, including ultrasound imaging, fluorescence imaging, or photoacoustic imaging, could enable visualization of tissue function, contractile force generation, and drug distribution without disrupting the system. For example, ultrasound imaging could be utilized to measure dynamic changes in muscle bundle thickness or contraction amplitude, while photoacoustic imaging might be used to monitor muscle contraction dynamics or detect localized changes in optical absorption within the biohybrid robot. We noted that advanced analytical technologies, such as those reported in recent studies, have already been applied in the biomedical field, achieving control and imaging of nano‐sized miniaturized robotics.^[^ ^42^, ^43^, ^44^, ^45^ ^]^ Given their proven utility in similarly complex biological systems, these technologies could be readily adapted to our platform, offering versatile tools to enhance physiological relevance and expand the analytical capabilities of our biohybrid robot‐on‐a‐chip. In conclusion, expanding the biohybrid robot‐on‐a‐chip system with multiple human‐derived organoid modules and advanced analytical tools will allow for more comprehensive modeling of complex human physiological processes. These enhancements will not only improve the platform's predictive accuracy for drug efficacy and toxicity but also position it as a promising alternative to animal models in biomedical research, ultimately contributing to the advancement of personalized medicine and regenerative therapies.

## Conflict of Interest

The authors declare no conflict of interest.

## Supporting information



Supporting Information

Supplemental Movie 1

Supplemental Movie 2

## Data Availability

The data that support the findings of this study are available from the corresponding author upon reasonable request.
