# Supplementary material for: Human Nervous System‐Based Biohybrid Robot‐On‐A‐Chip with Sensing Function for Toxicity Screening
Source: Adv Sci (Weinh). 2025 Jul 2;12(37):e01452. doi: 10.1002/advs.202501452 (PMC12499486; doi:10.1002/advs.202501452)

**SUPPORTING INFORMATION**

**Human nervous system-based biohybrid robot-on-a-chip with sensing function for toxicity test**

*Minkyu Shin^1,†^, Joungpyo Lim^1,†^, Seewoo Kim^1^, Sangeun Lee^1^, Wei Wen Su^2^, Jinho Yoon^3,*^, Jeong-Woo Choi^1,*^*

^1^Dr. M. Shin, Dr. J. Lim, Mr. S. Kim, Ms. S. Lee, Prof. J. -W. Choi

Department of Chemical & Biomolecular Engineering, Sogang University, 35 Baekbeom-ro, Mapo-gu, Seoul 04107, Republic of Korea

E-mail: jwchoi@sogang.ac.kr

^2^Prof. W. W. Su

Department of Molecular Biosciences and Bioengineering, University of Hawaii at Manoa, Honolulu, HI 96822, USA

^3^Prof. J. Yoon

Department of Biomedical-Chemical Engineering, The Catholic University of Korea, 43 Jibong-ro, Wonmi-gu, Bucheon-si, Gyeonggi-do 14662, Republic of Korea

E-mail: jyoon@catholic.ac.kr

† These authors contributed equally to this work as the first authors.

**TABLE OF CONTENTS FOR SUPPORTING INFORMATION**

1. **METHODS**
2. **SUPPLEMENTARY FIGURES**

**Figure S1** Generation of retinal organoids and thalamic organoids. A) Schematic illustration of generation of retinal organoids and thalamic organoids using the iPSCs. B) The generation process of retinal organoids. C) The generation process of thalamic organoids.

**Figure S2** Electrophysiological signals of the retinal organoid on the MEA. The yellow arrow means light stimulation.

**Figure S3** Synthesis and confirmation of the Au nanomesh. A) Schematic illustration of synthesis process of the Au nanomesh. B, C) FE-SEM images of PVA nanofiber. D, E) FE-SEM images of Au nanomesh.

**Figure S4** Confirmation of the biocompatibility of the Au nanomesh. A) Thalamic organoids stained with cleaved caspase-3 (cCasp3). B) Quantification of TNF-α levels in the thalamic organoid and Au nanomesh-encapsulated thalamic organoids.

**Figure S5** Confirmation of electrophysiological signal of thalamic and Au nanomesh-encapsulated thalamic organoid. Electrophysiological signals of the A) thalamic organoid and B) Au nanomesh-encapsulated thalamic organoid.

**Figure S6** Confirmation of effects of Au nanomesh for the generation of eye assembloid. A) Immunostaining images of the eye assembloid fabricated without Au nanomesh. Electrophysiological signals of the eye assembloid B) fabricated with Au nanomesh and C) fabricated without Au nanomesh.

**Figure S7** Confirmation of synthesis of the Au/MNPs. A) UV-Vis analysis of MNPs and Au/MNPs. B) Zeta potential analysis of Au/MNPs and mPEG modified Au/MNPs.

**Figure S8** Confirmation of the biocompatibility of the A/MNPs. A) Live and dead image. B) Quantification of dead cells.

**Figure S9** Design of solenoid coil for electromagnetic field stimulation. A) Design of the sinusoidal EMF device. B) Simulation image for EMF stimulation to cerebral organoid.

**Figure S10** Electrophysiological signal in the Au/MNP-incorporated cerebral organoid and control group.

**Figure S11** Confirmation of area of the Au/MNPs-incorporated cerebral organoid. A) Optical images of cerebral organoid and Au/MNPs-incorporated cerebral organoid. B) Quantification of area of the Au/MNPs-incorporated cerebral organoid.

**Figure S12** Confirmation of connections between the eye assembloid, cerebral organoid, and motor neuron spheroid. A) Optical and B) Confocal images of the connection of each organoid. B) Optical images of human nervous system-based biohybrid robot-on-a-chip.

**Figure S13** Fabrication of 3D printed chip. A) Design of 3D printed chip. B) Optical images of the 3D printed chip.

**Figure S14** Dose-response relationship analysis of different concentrations of hydroxychloroquine (HCQ) by the human nervous system-based biohybrid robot-on-a-chip.

**Table S1** List primers for qPCR.

**Movie S1** Muscle contraction of human nervous system based-biohybrid robot-on-a-chip with light stimulation.

**Movie S2** Toxicity test

**A. METHODS:**

**iPSC culture**: Two human iPSC lines, NCRM5AS1 and 1383D6, were used in this study. Both lines were verified to have normal karyotypes and were free of contamination. iPSCs were cultured on iMatrix-511 silk-coated plates with StemMACS medium, following established protocols. Cells were passaged every 5-7 days at 80% confluence. Colonies with clearly visible differentiated cells were marked and mechanically removed before passage.

**Generation of retinal organoid**: Embryonic bodies (EBs) were generated from iPSCs by seeding 1.0 × 10^4^ cells/well in ultra-low attachment round-bottom 96-well plates with StemMACS medium containing 50 μM of Rho-associated protein kinase (ROCK) inhibitor (Y-27632 dihydrochloride) for 24 h. After 24 h, EBs were gradually transitioned into neural induction medium (NIM) containing DMEM/F12 (1:1), 1% N2 supplement, 1 × nonessential amino acids (NEAAs), and 2 mg/mL heparin. The medium was gradually transitioned to 100% NIM over 3 days: a 3:1 ratio of StemMAC medium/NIM on day 1, 1:1 on day 2, and 100% NIM on day 3. On day 7, EBs were plated onto growth-factor-reduced Matrigel-coated dishes containing NIM and, on day 16, switched to DMEM/F12 (3:1) supplemented with 2% B27 (without vitamin A), 1 × NEAA and 1% antibiotic–antimycotic. Afterward, the medium was changed daily. On the fourth week of differentiation, Horseshoe-shaped neural retina domains were carefully detached using a sharpened tungsten needle under an inverted microscope. These detached domains were collected and cultured in suspension within a humidified 5% CO2 incubator. The culture medium consisted of DMEM/F12 (in a 3:1 ratio), supplemented with 2% B27, 1× NEAA, and 1% antibiotic-antimycotic. Over time, these conditions led to the gradual formation of 3D retinal cups. The medium was then changed every 3-4 days. For long-term suspension culture, the medium was supplemented with 10% fetal bovine serum, 2% GlutaMAX, and 100 mM taurine on day 42.

**Generation of thalamic organoid**: EBs were generated from iPSCs by seeding 1.0 × 10⁴ cells/well in ultra-low attachment round-bottom 96-well plates with StemMACS medium containing 50 μM ROCK inhibitor for 24 h. After 24 h, EBs were transitioned into an induction medium consisting of DMEM/F12 (1:1), 15% KnockOut Serum Replacement (KSR), 1 × NEAA, 1% GlutaMAX, and 100 mM β-mercaptoethanol, supplemented with 100 nm LDN-193189, 10 mM SB-431542, 4 mg/mL insulin, and 50 mM ROCK inhibitor. From day 2 onward, the medium was changed every 2 days. On day 8, EBs were transferred to spinning culture (80 rpm) in ultra-low-attachment 24-well plates. From day 8 to day 16, patterning media composed of DMEM-F12, 0.15% dextrose, 100 mM β-mercaptoethanol, 1% N2 supplement, and 2% B27 supplement (without vitamin A) was used. This medium was supplemented with 30 ng/mL BMP7 and 1 mM PD325901. The media was changed every other day during this period. On day 16, a differentiation medium was prepared as a 1:1 mixture of DMEM-F12 and Neurobasal media. The medium included the following supplements: 0.5% N2 supplement, 1% B27 supplement, 0.5% non-essential amino acids (NEAA), 1% GlutaMAX, 0.025% insulin, 50 mM β-mercaptoethanol, and 1% penicillin/streptomycin. Additionally, it was supplemented with 20 ng/mL of brain-derived neurotrophic factor (BDNF) and 200 mM ascorbic acid. The medium was changed every other day until day 25, and then it was changed every 4 days.

**Fabrication of Au nanomesh**: A PVA nanomesh was prepared using the electrospinning technique. First, 1.5 g of PVA powder was dissolved in 13.5 mL of distilled water at 70 °C for 2 h. The solution was then allowed to react overnight at room temperature to create a 10 wt% PVA aqueous solution. This PVA solution was filled into a 20 mL syringe and fed into an electrospinning apparatus. The distance between the syringe needle tip and the fiber collector was set to 30 cm. The PVA was electrospun onto a silicone-coated paper substrate to create a mesh structure. A voltage of 20 kV was applied while the PVA aqueous solution was ejected at a rate of 5 μL/min for 45 minutes to prepare the nanomesh sheet. A silicone-coated paper was placed on the fiber collector for easy delamination of the fabricated nanomesh sheet. The syringe needle used for electrospinning was metal with an inner diameter of 0.31 mm. The PVA solution had an average polymerization degree of 1,500–1,800 and a saponification degree of 78–82 mol%. Then, an Au layer was deposited on the surface of the PVA nanomesh sheet using vacuum deposition. To effectively attach nanomesh sheets to thalamic organoids, the nanomesh was formed by soaking the PVA-Au structure in water or culture medium to dissolve the PVA before introducing it to the thalamic organoids. Following this, dry the attached Au nanomesh at room temperature for 1 min. The fabrication process was confirmed using field emission scanning electron microscopy (FE-SEM).

**Generation of eye assembloid**: To generate the retina/thalamus assembloid, an alginate-based sacrificial hydrogel was used. First, the Au nanomesh-encapsulated thalamic organoid was placed in an alginate-based solution, and four retinal organoids were positioned near the Au nanomesh-encapsulated thalamic organoid using a pipette. Then, a tungsten needle was used to precisely attach the retinal organoids to the desired location on the Au nanomesh-encapsulated thalamic organoid. The alginate was subsequently sprayed with a calcium ion solution to induce gelation. The retinal organoid-attached Au nanomesh-encapsulated thalamic organoid was then incubated in an incubator for 3 days. During this time, the gelled alginate hydrogel spontaneously disintegrated, forming the eye assembloid.

**Synthesis of Au/MNPs**: The ZnFe_2_O_4_ nanoparticles (MNPs) were synthesized using the thermal decomposition method to serve as a template for the subsequent coating and growth of Au nanoparticles (AuNPs). To initiate the process, 1.41 g of iron (III) acetylacetonate (Sigma-Aldrich) and 0.267 g of zinc (II) chloride (Sigma-Aldrich) were dissolved in 40 mL of trioctylamine (Daejung chemical) buffer with 5.16 g of 1,2-hexadecanediol (TCI Chemical), 3.86 mL of oleylamine (Daejung chemical), and 3.82 mL of oleic acid (Sigma-Aldrich). The mixture was stirred rapidly (> 800 rpm) in a perfectly dried three-neck round-bottom flask for 2 h at 200 °C. Subsequently, the mixture was heated to 300 °C at a rate of 3 °C/min, and the reaction was maintained for 1 h at 300 °C while continuing rapid stirring. The reacted mixture was then allowed to cool to room temperature after the 1 h reaction, and the resulting MNP solution was collected once the temperature dropped below 30 °C.

To wash the MNPs, 30 mL of ethanol containing 1.92 mL of toluene was added to the collected MNPs solution, which was then centrifuged at 1,600 × g for 5 min at room temperature. The resulting supernatant was discarded, and the black precipitate was re-dispersed in 8 mL of toluene with 30 μL of oleylamine. The re-dispersed solution was centrifuged at 650 × g for 3 min at room temperature, and the supernatant was collected. Finally, 4 mL of ethanol was added to the supernatant and the mixture was centrifuged at 1,600 rpm for 5 min at room temperature. The precipitate was collected, re-dispersed in hexane, and stored at room temperature.

The surface of the MNPs was encapsulated with a silica layer using the standard water-in-oil microemulsion method. First, 616 μL of Igepal-CO 520 (Sigma-Aldrich) was dissolved in 12.6 mL of cyclohexane (Daejung chemical) for 30 min at 300 rpm. Then, 100 μL of the MNP solution (10 mg/mL in cyclohexane) was added to the mixture and stirred for an additional 30 min. For the surface coating of the silica layer, 140 μL of ammonia solution was added to the mixture, and the mixture was stirred until it became clear. Next, 15 μL of tetraethyl orthosilicate (TEOS) (Sigma-Aldrich) was added, and the reaction was stirred at 300 rpm for 48 h at room temperature. Following the reaction, the solution was washed three times with 10 mL of ethanol at 10,000 rpm for 10 min. The precipitate was re-dispersed in 30 mL of distilled water. Finally, 25 μL of APTES (Sigma-Aldrich) was added dropwise to the solution and stirred (300 rpm) overnight at room temperature. The prepared product, SiO_2_/MNPs, was then centrifuged at 10,000 rpm for 10 min and washed three times with 10 mL of ethanol.

To synthesize Au/MNPs, the prepared SiO_2_/MNPs were reacted with the Au seed solution. The Au seed solution was prepared by mixing 0.5 mL of 1 M NaOH, 1 mL of 1% tetrakis(hydroxymethyl)phosphonium chloride (THPC) (Sigma-Aldrich), and 38 mL of distilled water at 300 rpm for 10 min. Subsequently, 2 mL of 1% HAuCl_4_·3H_2_O (Sigma-Aldrich) was added, and the reaction was continued at 300 rpm for 2 min. The resulting Au_seed_/SiO_2_/MNPs were collected by centrifugation at 10,000 rpm for 10 min, washed with distilled water to remove the extra Au seeds, and re-dispersed in 1 mL of distilled water. To grow the Au layer on the surface of the Au_seed_/SiO_2_/MNPs, 720 μL of 0.5% PVP solution (Sigma-Aldrich) containing 30 mL of K_2_CO_3_/HauCl_4_ and 720 μL of 20 mM NH_2_OH·HCl solution was added, and the mixture was stirred at 300 rpm for 10 min at room temperature. The synthesized Au/MNPs were collected using neodymium magnets and stored in ethanol solution at 4 ℃.

To enhance the biocompatibility of the Au/MNPs, their surfaces were modified with mPEG-SH (Sigma-Aldrich). Briefly, 2 mL of 0.5 mg/mL mPEG-SH was mixed with 2.5 mg/mL of Au/MNPs, and the mixture was stirred for 30 min at 60 ℃. After modification, the Au/MNPs were centrifuged at 7,000 rpm for 30 min, washed three times with distilled water and ethanol, and then collected.

**Generation of Au/MNP-incorporated cerebral organoid**: EBs were generated from iPSCs by seeding 1.0 × 10^4^ cells/well in ultra-low attachment round-bottom 96-well plates (SPL Life Science) with StemMACS medium containing 50 μM ROCK inhibitor (Y-27632 dihydrochloride, Biogems) and 4 ng/mL basic fibroblast growth factor (bFGF) (Peprotech) for 24 h. After 24 h, EBs were formed, and the medium was replaced with fresh StemMACS medium containing 4 ng/mL bFGF and 50 μM ROCK inhibitor. On day 4, the medium was switched to StemMACS medium containing 2 ng/mL bFGF. On day 5, the EBs were transferred to a 60 mm ultra-low attachment Petri dish. From day 5 to day 9, the medium was changed every 2 days to DMEM/F-12 (WELGENE) supplemented with 1 × N2 supplement (Thermo Fisher Scientific), 1 × GlutaMAX supplement (Gibco), 1 × MEM-NEAA solution (Sigma-Aldrich), and 1 μg/mL of heparin (Sigma-Aldrich) to promote neuroepithelium-like tissue formation. On day 9, each neuroepithelial tissue was embedded in Matrigel (Corning) droplets containing 25 μg/mL of Au/MNPs (10 μL) on a sterilized 60 mm dish with an embedding sheet (STEMCELL Technologies) and incubated at 37 ℃ for 30 min to promote polymerization of Matrigel. Subsequently, 8 mL of a 1:1 mixture of DMEM/F-12 and Neurobasal media (Thermo Fisher Scientific) with 1:200 (v/v) N2 supplement, 1:100 (v/v) B-27 supplement (without vitamin A, Gibco), 1:100 (v/v) GlutaMAX supplement, 1:200 (v/v) MEM-NEAA solution, 50 μM 2-mercaptoethanol (Gibco), and 2.5 μg/mL insulin (Sigma-Aldrich) was prepared. Next, 100 μg/mL streptomycin (Gibco) and 100 U/mL penicillin were added sequentially. The Matrigel was detached from the embedding sheet and the medium was changed every 2 days. On day 13, the dish with cerebral tissues was placed on an orbital shaker, and the maturation medium was changed every 3 days with a 1:1 mixture of DMEM/F-12 and Neurobasal media containing 1:200 (v/v) N2 supplement, 1:200 (v/v) MEM-NEAA solution, 1:100 (v/v) GlutaMAX supplement, 2.5 μg/mL insulin, 50 μM 2-mercaptoethanol, and 1:100 (v/v) B-27 supplement (Gibco). The medium was also supplemented with 100 μg/mL and 100 U/mL penicillin. On day 20, the cerebral tissue was placed in the center of the EMF device, and EMF stimulation (60 Hz, 2 mT) was applied for 1 h per day for 1 week.

**Design of solenoid coil and EMF stimulation**: To apply uniform EMF stimulation to the cerebral organoids, an insulated copper wire was tightly wound around a cylindrical polyethylene tube (6.5 cm diameter, 20 cm length) and connected to an electrical supply. The wire was designed with a resistance of 650 Ω to generate a magnetic flux density of 2 mT when powered. A 60 Hz sinusoidal wavelength generator was linked to the power supply to create the desired EMF. The setup was placed in a 37 ℃ incubator with a humidified atmosphere of 5% CO_2_, and EMF stimulation (60 Hz, 2 mT) was applied daily for over 7 days. Furthermore, to assess the effects of EMF and Au/MNPs on the neurogenesis of cerebral organoids, three experimental groups were prepared: (i) control (without EMF), (ii) control (with EMF), and (iii) 25 μg/mL (with EMF).

**Generation of motor neuron spheroid**: Human neural stem cells (NSCs) were cultured on laminin-coated tissue culture plates with StemPro Neural supplement (Gibco) (20 μL/mL), bFGF (20 ng/mL), KnockOut DMEM/F-12 medium (Gibco), ascorbic acid (200 μM, Sigma-Aldrich), GlutaMAX supplement (2 mM), heparin (6 units/mL), and EGF (20 ng/mL, Peprotech). NSCs (5.0 ×10^4^ cells/well) were seeded in round-bottomed ultra-low attachment 96-well plates to form neural spheroids. After 24 h, the culture medium was replaced with a mixture of StemPro hESC medium (Gibco), 10 ng/mL Activin A (Peprotech), 50 μM retinoic acid (Sigma-Aldrich), 8 ng/mL of bFGF, and 200 ng/mL of sonic hedgehog (Peprotech) to induce motor neuron differentiation. After 20 days, the medium was changed to StemPro hESC (Gibco) supplemented with 10 ng/mL GDNF (Peprotech) and 10 ng/mL BDNF (Peprotech) for 8 days to mature the motor neurons.

**Fabrication of human nervous system-based biohybrid robot-on-a-chip**: To fabricate the human nervous system-based biohybrid robot-on-a-chip, a 3D printed chip was designed using 3D MAX and fabricated using a Stereolithography (SLA) 3D printer (Formlabs 4). The bottom of the 3D printed chip was bonded to polydimethylsiloxane (PDMS) (Dow Corning) and cured at 70 cc for 2 h. To immobilize the muscle bundle on the PDMS, pillars made from Ecoflex 00-30 were attached to the PDMS. The chip was then washed with ethanol and distilled water and sterilized with UV light for 2 h. First, the differentiated eye assembloid and cerebral organoid were positioned on the 3D-printed chip. Next, muscle bundles were transferred and connected to the motor neuron spheroids using Matrigel. The human nervous system-based biohybrid robot-on-a-chip was developed by co-culturing the eye assembloid and cerebral organoid in maturation medium in Channel 1, and the motor neuron spheroid and muscle bundle in muscle bundle differentiation medium supplemented with 10 ng/mL of glial cell line-derived neurotrophic factor (GNDF) and 10 ng/mL of BDNF in Channel 2 for 7 days. After co-culturing in the maturation medium, the human nervous system-based biohybrid robot-on-a-chip was complete.

**Immunohistochemistry**: For immunostaining analysis, organoids were fixed in 4% formaldehyde for 3 h at 20 ℃. Following three washes with Dulbecco’s phosphate-buffered saline (DPBS), the fixed samples were permeabilized by immersing in 0.2% Triton-X solution for 60 min and subsequently blocked with 2% BSA for 30 min. For the eye organoid, samples were incubated overnight at 4 °C with the following primary antibodies: rabbit monoclonal anti-TCF7L2 (1:1000, MA5-14975, Invitrogen), rabbit monoclonal anti-L/M opsin (L/M opsin) (1:1000, AB5405, Merck), and mouse monoclonal anti-rhodopsin (Rhodopsin) (1:1000, MABN15, Merck). After culturing with primary antibodies, samples were washed with DPBS and treated with secondary antibodies. For the cerebral organoid, samples were incubated overnight at 4 °C with the following primary antibodies: anti-SOX2 (1:50, ab93689, Abcam) and anti-PAX6 (1:50, sc-81649, Santa Cruz Biotechnology). Following this, the samples were incubated for 3 days at 25 ℃. The samples were then washed with DPBS and incubated with secondary antibodies [(FITC-modified IgG (1: 100) and Texas Red-modified IgG (1:100)] for 2 days. The nuclei of the cerebral organoids were stained with Hoechst (3 µg/mL) for 3 min. Immunostaining was analyzed using confocal microscopy (LSM 710, Carl Zeiss, Jena, Germany).

**qPCR analysis**:

To confirm the differentiation of cells into specific organoids, gene expression was assessed using qPCR analysis. Genomic RNA was isolated from each sample using TRIzol reagent. Reverse transcription was performed using AccuPower CyclerScript RT PreMix according to the manufacturer's protocol. Quantitative PCR analysis was conducted using an Exicycler 96 system (Bioneer) with AccuPower Taq PCR PreMix. The expression level of GAPDH, a housekeeping gene, was used for normalization across all samples. We utilized the expression level of GAPDH, a key housekeeping gene, for normalization across all samples, ensuring consistent and reliable results.

**Evaluation of electrophysiological activity**: Electrophysiological signals from the organoids were recorded using the MEA system from Axion BioSystems. The 16-electrode arrays of the MEA plate were pre-coated with 0.1% PEI and laminin for 60 min and 3 h at 37 ℃, respectively. Each organoid was placed on the MEA plate, and electrophysiological signals were recorded at a sampling rate of 12.5 kHz for 5 min at 37 ℃ in the appropriate culture medium. The Butterworth bandpass filter was applied with cutoff frequencies set between 200 Hz and 3,000 Hz, along with a threshold of six times the standard deviation (SD) to reduce false positive signals. This process was carried out using the Axion Integrated Studio program. The resulting spike raster graphs were then analyzed using the Neural Metric Tool from Axion BioSystems.

**Light stimulation**: The pE-4000 illumination system (CoolLED, UK), an LED-based light source, was employed for light stimulation at wavelengths of 435 nm, 500 nm, 550 nm, and 660 nm. Photocurrent measurements were performed in a dark room to minimize interference from external light sources, ensuring precise and reliable results.

**B. SUPPLEMENTARY FIGURES AND TABLES:**


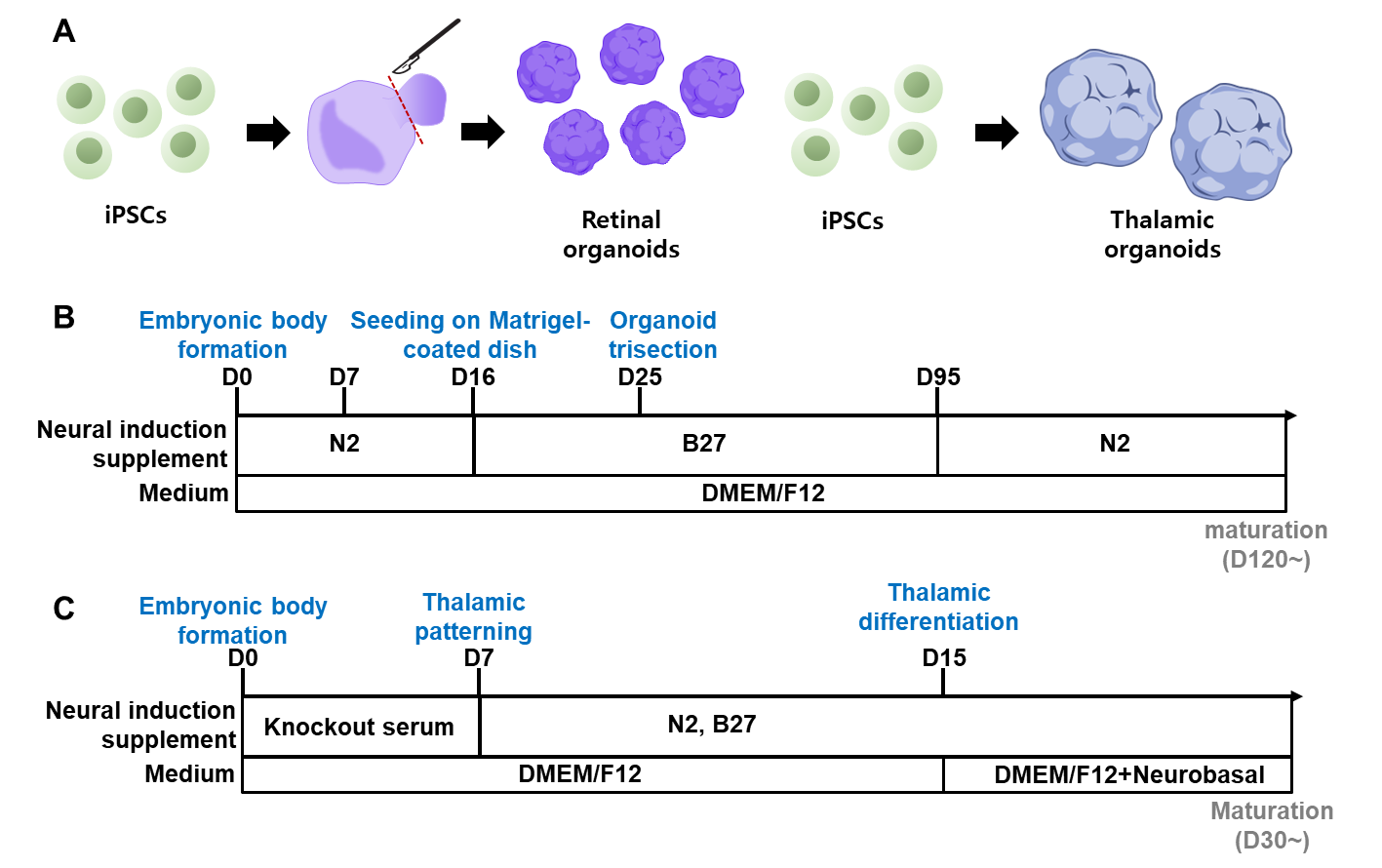


**Figure S1.** **Generation of retinal organoids and thalamic organoids.** A) Schematic illustration of generation of retinal organoids and thalamic organoids using the iPSCs. B) The generation process of retinal organoids. C) The generation process of thalamic organoids.


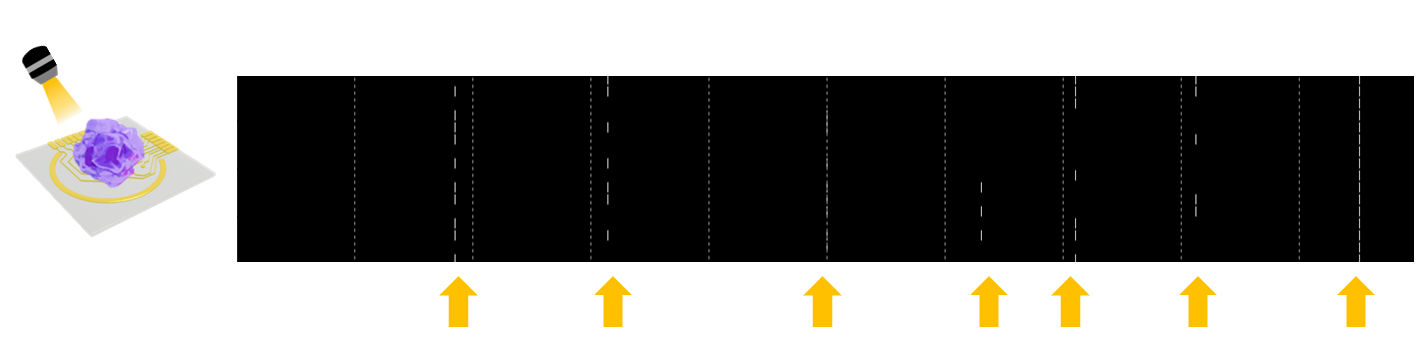


**Figure S2.** **Electrophysiological signals of the retinal organoid on the MEA.** The yellow arrow means light stimulation.


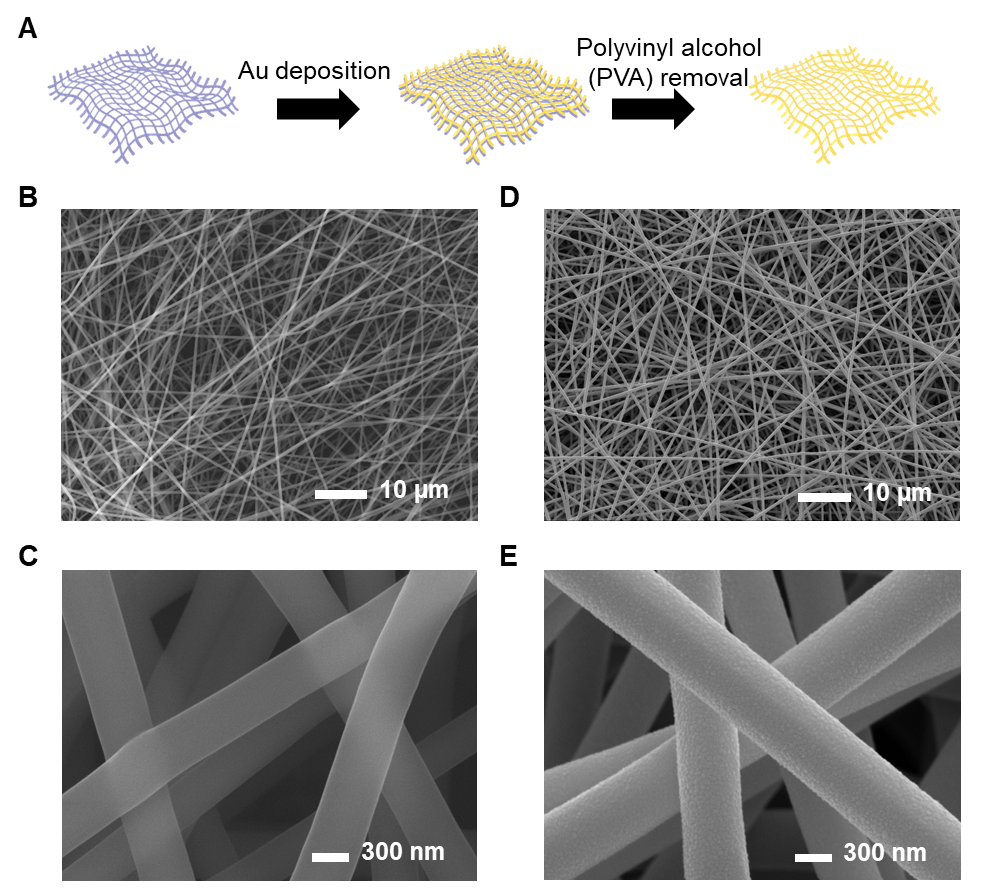


**Figure S3.** **Synthesis and confirmation of the Au nanomesh.** A) Schematic illustration of synthesis process of the Au nanomesh. B, C) FE-SEM images of PVA nanofiber. D, E) FE-SEM images of Au nanomesh.

**
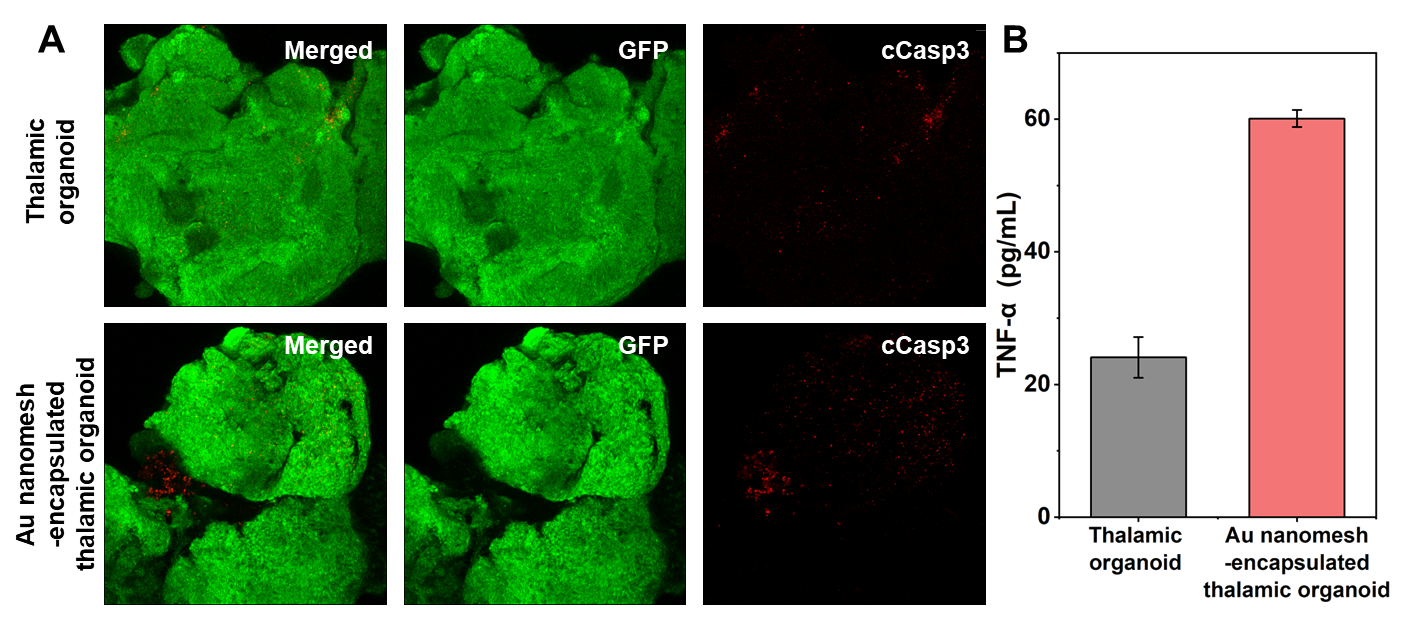
**

**Figure S4. Confirmation of the biocompatibility of the Au nanomesh.** A) Thalamic organoids stained with cleaved caspase-3 (cCasp3). B) Quantification of TNF-α levels in the thalamic organoid and Au nanomesh-encapsulated thalamic organoids.


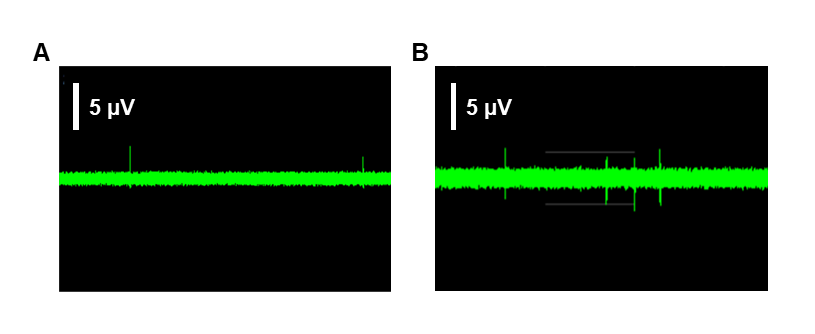


**Figure S5.** **Confirmation of electrophysiological signal of thalamic and Au nanomesh-encapsulated thalamic organoid.** Electrophysiological signals of the A) thalamic organoid and B) Au nanomesh-encapsulated thalamic organoid.


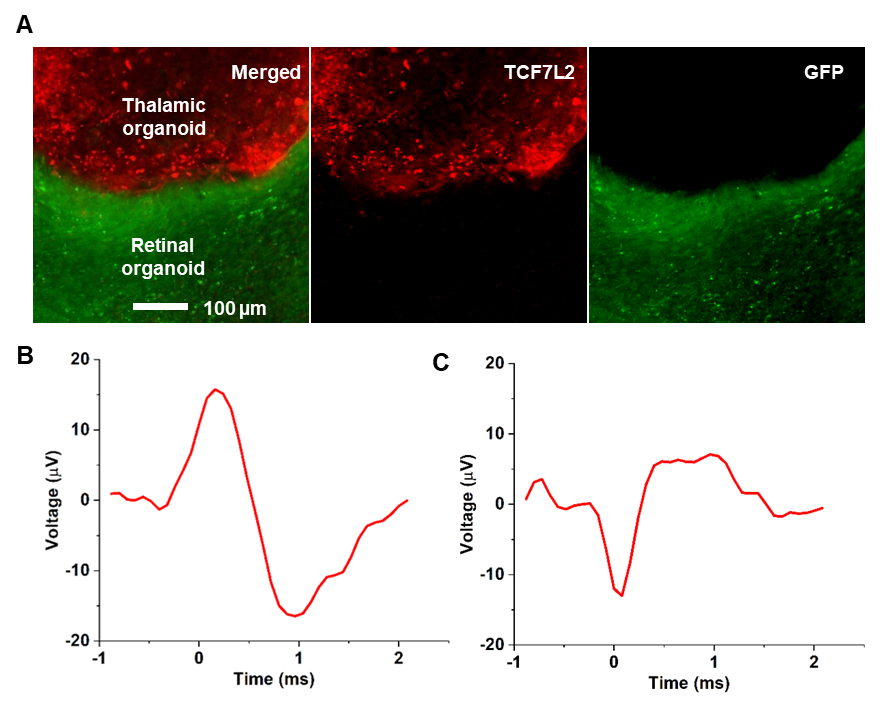


**Figure S6.** **Confirmation of effects of Au nanomesh for the generation of eye assembloid.** A) Immunostaining images of the eye assembloid fabricated without Au nanomesh. Electrophysiological signals of the eye assembloid B) fabricated with Au nanomesh and C) fabricated without Au nanomesh.


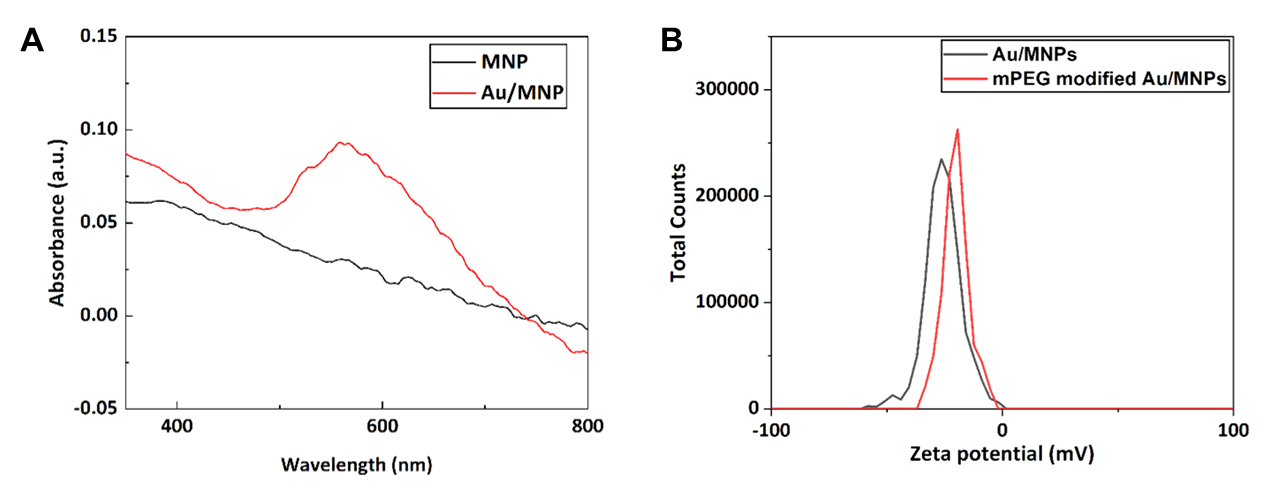


**Figure S7.** **Confirmation of synthesis of the Au/MNPs.** A) UV-Vis analysis of MNPs and Au/MNPs. B) Zeta potential analysis of Au/MNPs and mPEG modified Au/MNPs.

**
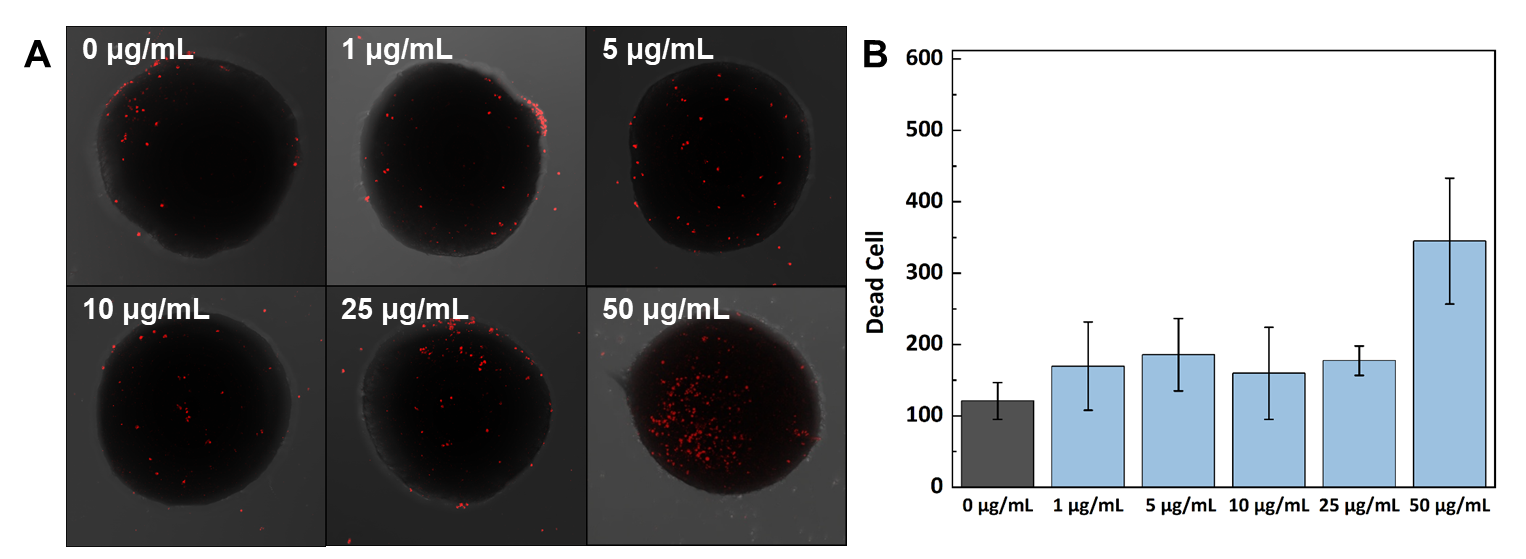
**

**Figure S8. Confirmation of the biocompatibility of the Au/MNPs.** A) Live and dead image. B) Quantification of dead cells.

**
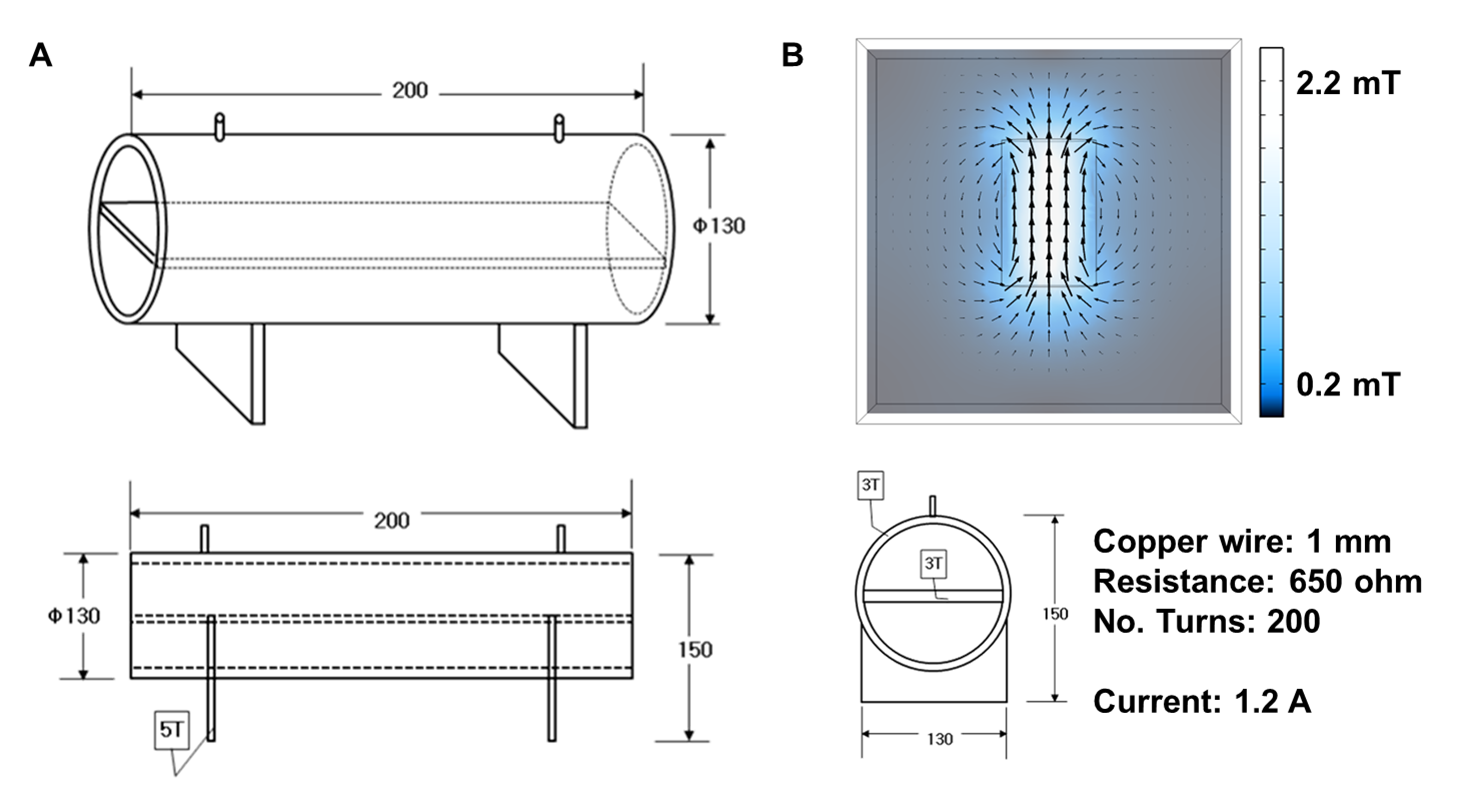
**

**Figure S9.** **Design of solenoid coil for electromagnetic field stimulation**. A) Design of the sinusoidal EMF device. B) Simulation image for EMF stimulation to cerebral organoid.

**
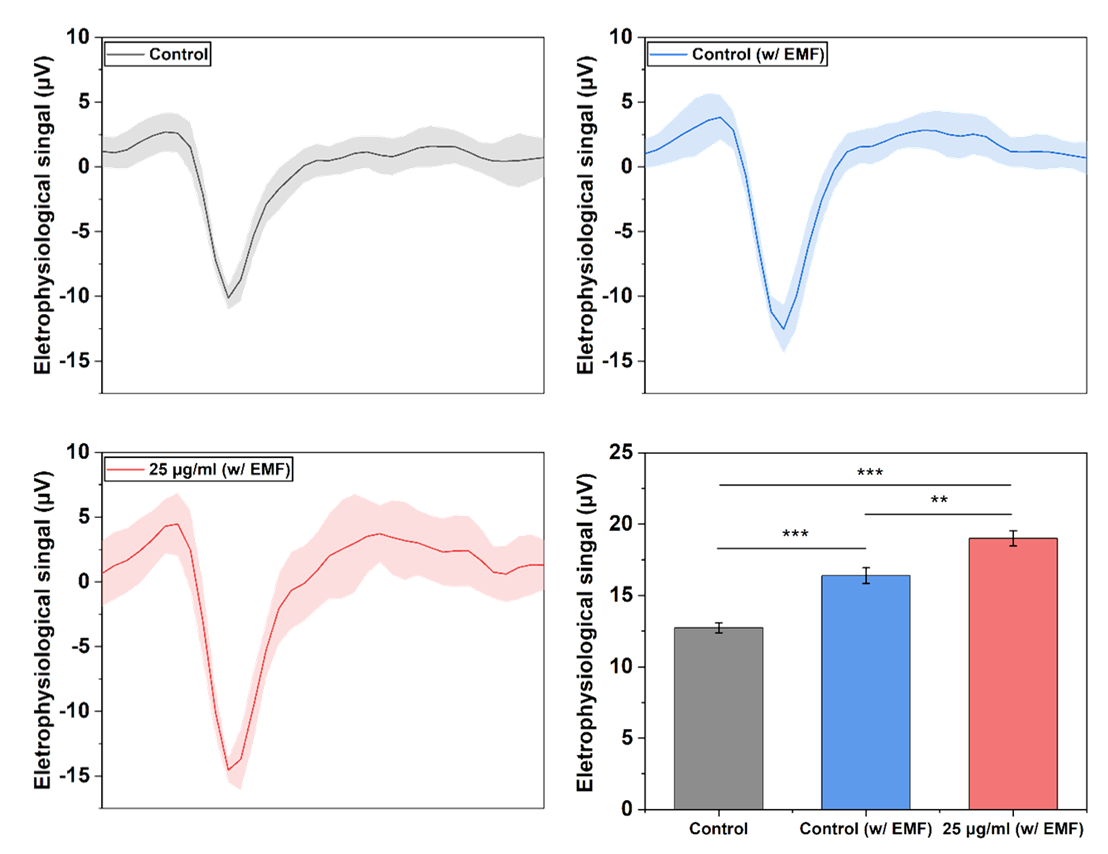
**

**Figure S10. Electrophysiological signal in the Au/MNP-incorporated cerebral organoid and control group.**

**
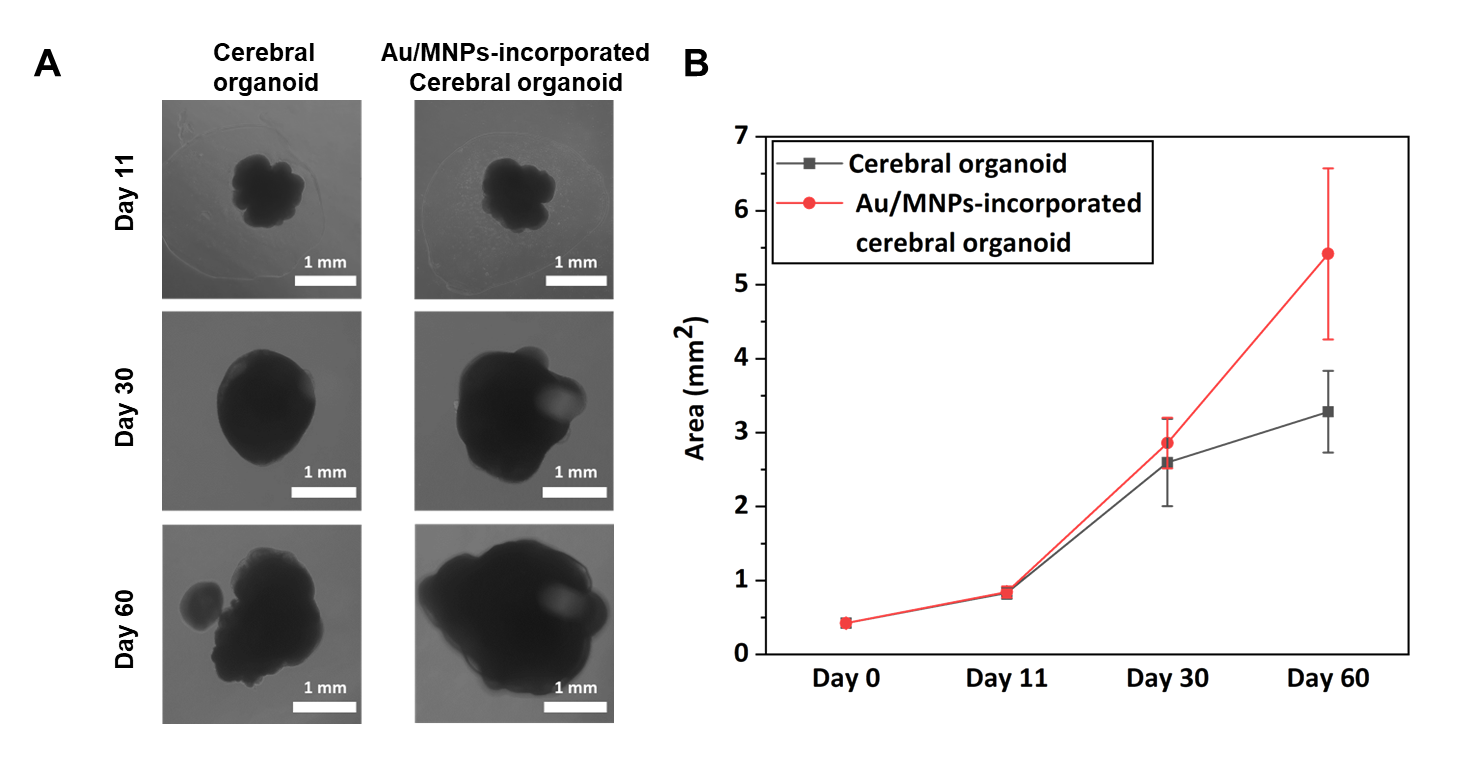
**

**Figure S11.** **Confirmation of area of the Au/MNPs-incorporated cerebral organoid.** A) Optical images of cerebral organoid and Au/MNPs-incorporated cerebral organoid. B) Quantification of area of the Au/MNPs-incorporated cerebral organoid.


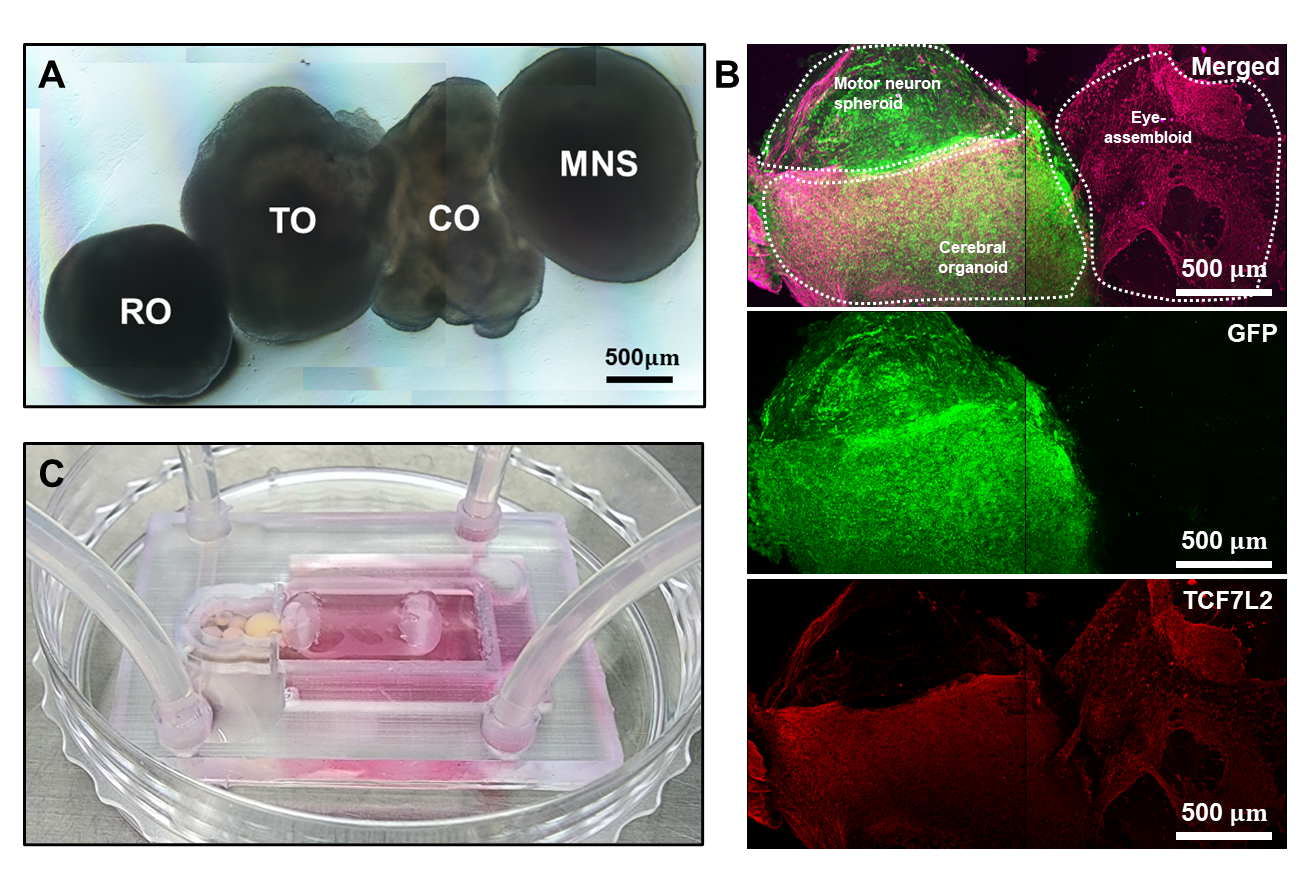


**Figure S12. Confirmation of connections between the eye assembloid, cerebral organoid, and motor neuron spheroid.** A) Optical and B) Confocal images of the connection of each organoid. B) Optical images of human nervous system-based biohybrid robot-on-a-chip.

**
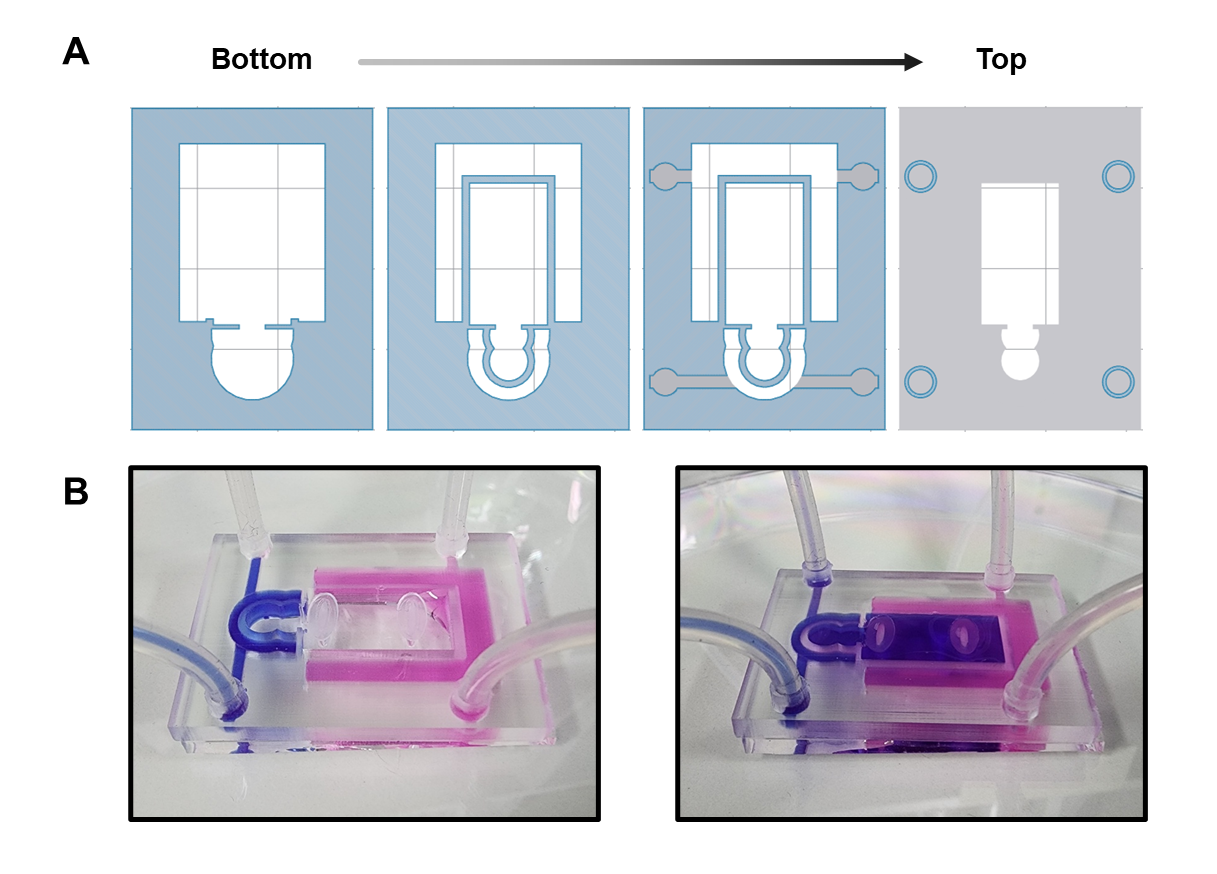
**

**Figure S13. Fabrication of 3D printed chip.** A) Design of 3D printed chip. B) Optical images of the 3D printed chip.

**
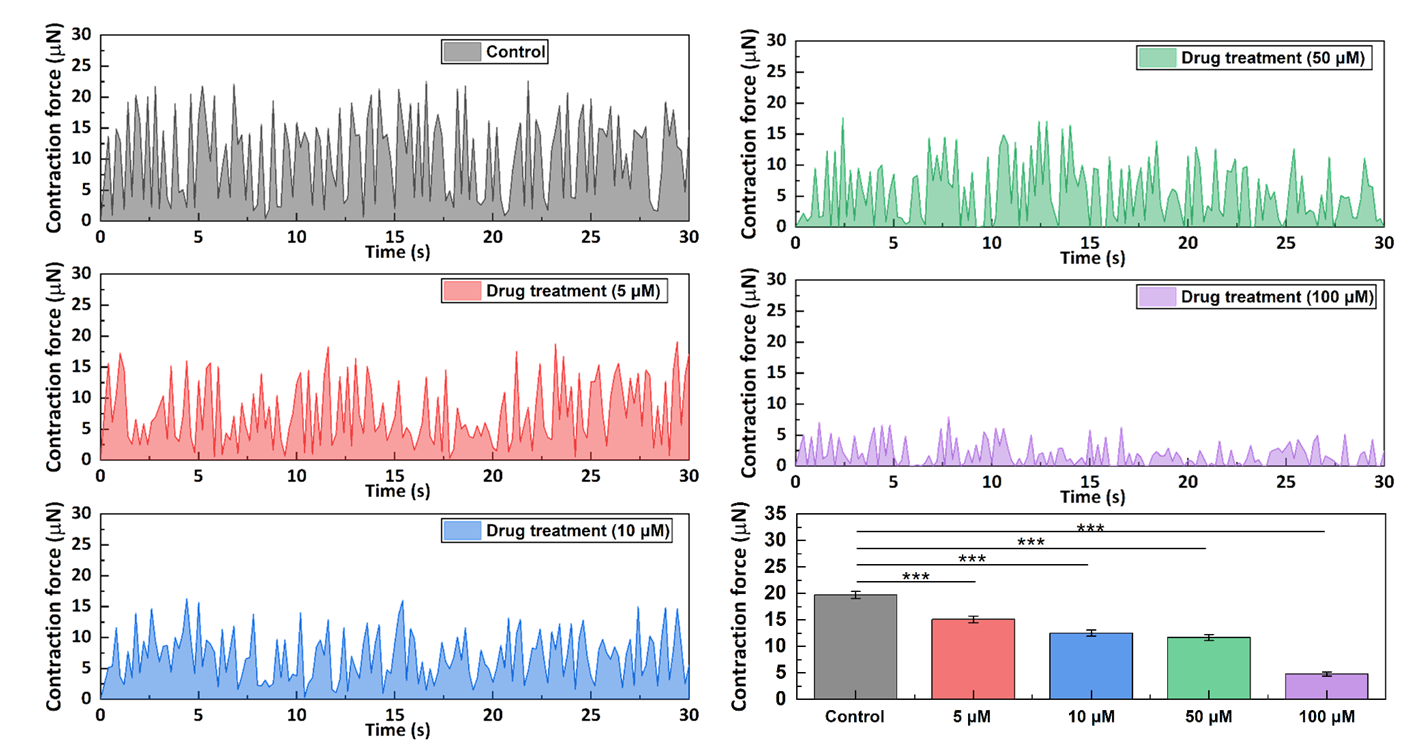
**

**Figure S14. Dose-response relationship analysis of different concentrations of hydroxychloroquine (HCQ) by the human nervous system-based biohybrid robot-on-a-chip.**

**Table S1. List primers for qPCR**


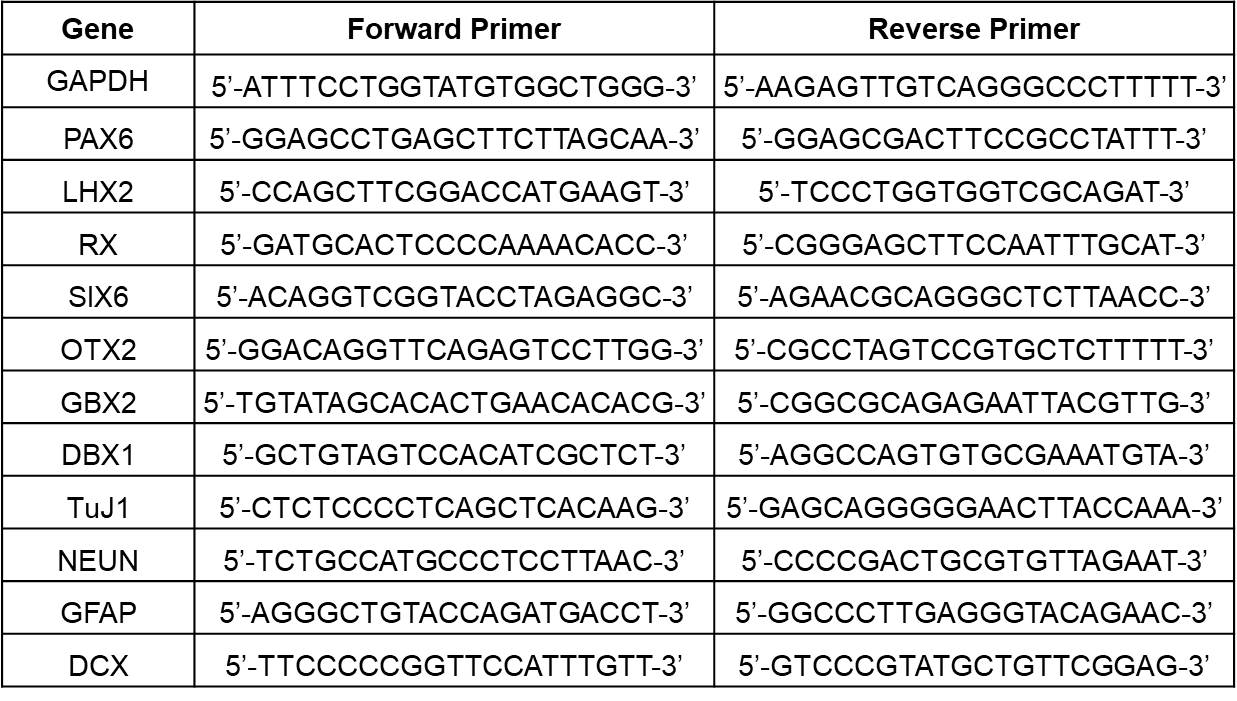

Supplement: Supplementary file 1 — Supporting Information [file ADVS-12-e01452-s002.docx]
